# Digital Phenotyping of Lifestyle Profiles and Mental Well-Being in German Adults: Prospective Longitudinal Cohort Study

**DOI:** 10.2196/101850

**Published:** 2026-09-14

**Authors:** Ningzhe Zhu, Ramona Schoedel, Larissa Sust, Markus Bühner, Yannik Terhorst

**Affiliations:** 1 Department of Psychology, Ludwig-Maximilians-Universität München, Leopoldstr. 13, Munich, Bavaria, 80802, Germany, 49 89 2180 5194; 2 University of Psychology, Charlotte Fresenius Hochschule, Munich, Bavaria, Germany; 3 German Center for Mental Health (DZPG), Partner-Site Munich-Augsburg, Bavaria, Germany

**Keywords:** Digital phenotyping, person-centered approach, mobile sensing, mental health, personality

## Abstract

**Background:**

Digital phenotyping uses passively collected smartphone-sensing data to characterize everyday behavior in naturalistic settings, and has become an important approach for studying mental well-being. Most previous studies have examined associations between individual sensing variables and mental health. However, mental well-being is likely reflected not by isolated behaviors but by combinations of co-occurring daily behaviors that together form lifestyles. Person-centered approaches capable of identifying these behavioral configurations may, therefore, provide more interpretable digital phenotypes; yet, such approaches have rarely been applied to passive smartphone-sensing data.

**Objective:**

This study aimed to examine whether smartphone-captured behavioral and environmental data could be used to derive interpretable day-level and person-level lifestyle profiles, and whether person-level profiles were associated with mental well-being. We also tested whether Big Five personality traits—extraversion, agreeableness, conscientiousness, openness, and negative emotionality—moderated these associations.

**Methods:**

The study used a 2-week prospective longitudinal cohort design with a sample of 553 German adults (mean age 42.12, SD 12.89 years; 44.65% female) drawn from an initial sample recruited according to quotas designed to reflect the German population. Ten smartphone-sensing indicators captured 5 domains, including communication and social media app use, mobility, physical activity, environmental context, and phone-use intensity. Mental well-being was assessed using the Warwick–Edinburgh Mental Well-Being Scale, and personality was assessed using the 15-item Big Five Inventory–2 Extra-Short Form. We used multilevel latent profile analysis to identify day-level profiles nested within person-level profiles. Associations between profiles and mental well-being were tested using classification-error–adjusted mean comparisons and omnibus Wald tests. Moderation was examined using hierarchical regressions comparing models with and without profile-by-personality interactions.

**Results:**

Eight day-level profiles and 7 person-level profiles were identified. Day-level profiles reflected distinct combinations of smartphone-sensing indicators. Person-level profiles represented different distributions of these daily patterns. Profiles differed significantly only in positive functioning (Wald *χ*²_6_=13.39; *P*=.04), not in overall mental well-being, positive affect, or satisfying interpersonal relationships. The physically active and unplugged profile had higher positive functioning than the mobile and always-on social profile (mean 3.94, SD 0.63 vs mean 3.61, SD 0.74; Cohen *d*=0.47; 95% CI 0.21‐0.73). No other pairwise differences were significant. Sensitivity analyses excluding the smallest profile produced comparable results, supporting the robustness of the findings. Personality-by-profile interactions did not significantly improve prediction for any well-being outcome.

**Conclusions:**

The findings extend the field by showing that transparent, person-centered digital phenotypes can distinguish variation in positive functioning, although causal conclusions cannot be drawn. In real-world settings, such interpretable profiles could support understandable monitoring tools and, following prospective replication and validation, inform personalized multibehavior interventions that target combinations of behaviors rather than single behaviors in isolation.

## Introduction

### Problem

As the World Health Organization (WHO) notes, mental well-being is “crucial to personal, community, and socioeconomic development” [1]. Mental well-being, or positive mental health, is more than just the absence of mental illness. It also includes having positive psychological resources [2]. Integrating hedonic and eudaimonic perspectives, researchers have highlighted 3 aspects of mental well-being, namely positive affect (eg, optimism, cheerfulness, and relaxation), positive functioning (eg, energy, clear thinking, and competence), and satisfying interpersonal relationships (a sense of belonging and being loved) [3]. Naturally, mental well-being fluctuates over time, and these dynamics typically manifest in daily life [4,5]. They are reflected in and can thus be inferred from everyday behavioral patterns [6]. Previous research in this area relied on self-reports and laboratory experiments [7-9]. However, self-reports are vulnerable to recall and social desirability biases [10], while laboratory paradigms lack ecological validity [11]. Mobile sensing, which passively collects usage records and sensor data from smartphones, helps overcome both limitations by continuously and unobtrusively sampling behavior, providing a more accurate reflection of people’s daily lives [12]. With smartphones now in the pockets of over 3‐quarters of the world’s population [13], capturing these fine‐grained contours of daily life [14,15] has become feasible, allowing us to access app‐use durations, GPS‐derived mobility, and ambient light or sound levels that may reflect well‐being [16,17].

### Review of Relevant Scholarship

To date, most work in digital mental health has pursued two primary paths: (1) correlational designs that link individual phone-use logs or sensor data to mental well-being or illness assessed by self-report measures or diagnostic interviews [18-27]; or (2) machine learning models aimed at maximizing predictive accuracy of mental health indicators from high‐dimensional sensor inputs [28-32]. While valuable, these strategies are primarily population-level because they often focus on average between-person relationships that can hide the rich heterogeneity of individual experiences [14,33]. In particular, correlational designs and many machine-learning pipelines, especially those trained on temporally aggregated features, can overlook how combinations of behaviors co-occur within the same day, information that is crucial for identifying well-being-related lifestyles and designing personalized interventions [34-36].

Recently, a growing consensus emerged that mental well-being is rarely driven by any single behavior in isolation; what matters are combinations of co-occurring behaviors and the ways they recur as patterns [37,38]. In other words, it is about the synergy, not the dose, of certain behaviors, and mental well-being is likely reflected in the patterned ensemble of behaviors [39]. For example, researchers have found that social engagement [40], mobility patterns [41], physical activity [42], phone use [43], and the environment [44] predict mental health, not in isolation but in combination. Measuring these indicators in real-world settings and using a person-centered modeling approach enables researchers to identify distinct daily behavioral patterns. Furthermore, by examining how individuals assemble and repeat these daily patterns, we can identify broader profiles of lifestyles [16,17] and see which lifestyles demonstrate better mental well-being.

A person-centered approach seeks to identify subpopulations that exhibit distinct patterns across certain variables [45]. Latent profile analysis (LPA) is particularly well suited to this purpose [46]. It is a statistical method that estimates “latent” (ie, not directly observed but inferred) profiles for each unit (eg, one day) and—based on these profiles—groups units that exhibit specific levels on observed measures [47]. LPA aims to identify groups of units that show similar patterns on these measures. For a detailed methodological illustration, see S1 in Multimedia Appendix 1. In digital phenotyping, selecting the unit to be profiled is consequential. A conventional single-level LPA would typically aggregate sensing indicators across the observation period and classify individuals according to their average or total levels of these indicators. Although this approach can identify broad between-person differences, aggregation removes information about how behaviors are organized within individual days and how different types of days recur within the same person. It may also introduce ecological fallacy if associations observed for person-level averages are interpreted as reflecting behavioral configurations occurring within individual days [48]. Two persons may have identical 2-week averages but arrive at those averages through different daily configurations. For example, one person may consistently display moderate mobility and phone use, whereas another alternates between highly active, socially engaged days and sedentary, digitally intensive days. A single-level analysis of aggregated indicators may place these individuals in the same profile, despite potentially meaningful differences in the regularity, variability, and composition of their lifestyles. Profiling days before profiling persons therefore preserves information that is lost when observations are immediately collapsed to person-level values. Usually, repeated digital traces (eg, app-use sessions) are nested within individuals, yielding a hierarchical structure: daily behavioral patterns (Level 1) [12] are nested within persons, and across days, these patterns aggregate into individual lifestyles (Level 2) [49]. A 2-level LPA can capture this structure well. At Level 1, multivariate sensing features from each day can be clustered into daily behavioral profiles; at Level 2, individuals can be clustered by their distribution across these daily profiles to yield higher-order personal profiles [50]. This hierarchical approach has been widely used across organizational, educational, affective, and psychopathological research and has proven effective for identifying subpopulations that differ in distal outcomes (eg, mental health and academic performance) [51-55]. However, to date, no study has applied this approach to identifying behavioral patterns captured via smartphones. By moving beyond variable-centered correlations and single-stage pipelines that operate at a single timescale (eg, collapsing days into person-level summary features before modeling or modeling daily events without considering person clusters), 2-level LPA yields interpretable lifestyles that may offer useful insights for future personalization efforts, positioning it to substantially advance digital mental health research.

Additionally, theory suggests that person-behavior fit plays a crucial role in well‐being. Individuals thrive when their activities align with their personalities [56,57]. For example, extraverts may flourish on highly social, connected days, whereas conscientious individuals might derive greater satisfaction from structured, goal‐driven routines [58,59]. Thus, personality traits may be integrated into such latent profile frameworks to test whether different personality traits are associated with mental well-being in the same way across different lifestyles and whether individuals with lower or higher levels of these traits exhibit different levels of mental well-being across profiles.

### Hypothesis, Aims, and Objectives

Implementing this approach, the present study used a 2-level LPA on 2 weeks of phone-use logs and sensor data capturing social engagement, mobility, physical activity, phone usage, and environment in a German adult sample. At Level 1, we uncovered recurring daily behavioral phenotypes; at Level 2, we identified personal profiles based on the distribution of daily profile proportions. The study had three objectives: (1) to determine whether passively collected smartphone-sensing data could be used to derive interpretable day-level and person-level lifestyle profiles; (2) to examine whether the resulting person-level profiles differed in overall mental well-being and its dimensions of positive affect, positive functioning, and satisfying interpersonal relationships; and (3) to test whether Big Five personality traits—extraversion, agreeableness, conscientiousness, openness, and negative emotionality—moderated these associations.

## Methods

### Participants and Settings

The data used in the current study were obtained from the Smartphone Sensing Panel Study (SSPS), a prospective longitudinal cohort study conducted by Ludwig-Maximilians-Universität München (LMU Munich) in cooperation with the Leibniz Institute for Psychology [60]. The SSPS collected data from May 2020 to November 2020. Participants were asked to install the self-developed Android-based mobile sensing app PhoneStudy (LMU Munich) on their private smartphones, which continuously collected various data in the background throughout the respective study duration. Online surveys, experience sampling, and smartphone-sensing data were collected.

The original SSPS aimed to recruit 800 participants. This target was determined pragmatically by balancing the planned study duration, expected attrition, and participant compensation costs. Based on an anticipated monthly dropout rate of 7%‐18.5%, approximately 430 participants were expected to remain after 6 months. Ultimately, 850 participants installed the application and participated in the study, of whom 750 completed the initial survey containing demographic information in May 2020. All procedures adhered to the General Data Protection Regulation. Reporting follows the STROBE (Strengthening the Reporting of Observational Studies in Epidemiology [61]) guidelines for observational studies.

### Inclusion and Exclusion

In the present study, we used questionnaire data from the July 2020 survey, which measured mental well-being and Big Five personality traits. A total of 636 participants completed this wave of questionnaires, from whom we extracted sensing data. We excluded 8 participants with no sensing records and 16 who changed phones during data collection, leaving 612 participants. Sensing data were extracted up to the day before each user filled out the questionnaire (July 12, 2020‐July 25, 2020). Because mental well-being was assessed from each participant’s subjective evaluation over the preceding 2 weeks, we first extracted the most recent 14 days of data for everyone. We then applied the 4/7 validity rule [62], which required at least 4 days of valid data within any 7-day window and corresponded to a minimum of 8 valid days over the 14-day period. A day was classified as invalid when all sensing variables were either zero or missing. Participants with fewer than 8 valid days were excluded, leaving a final sample of 553 individuals (mean age 42.12, SD 12.89 years; 44.65% female) with 7635 days of sensing data.

### Participant Characteristics

**Figure 1.**
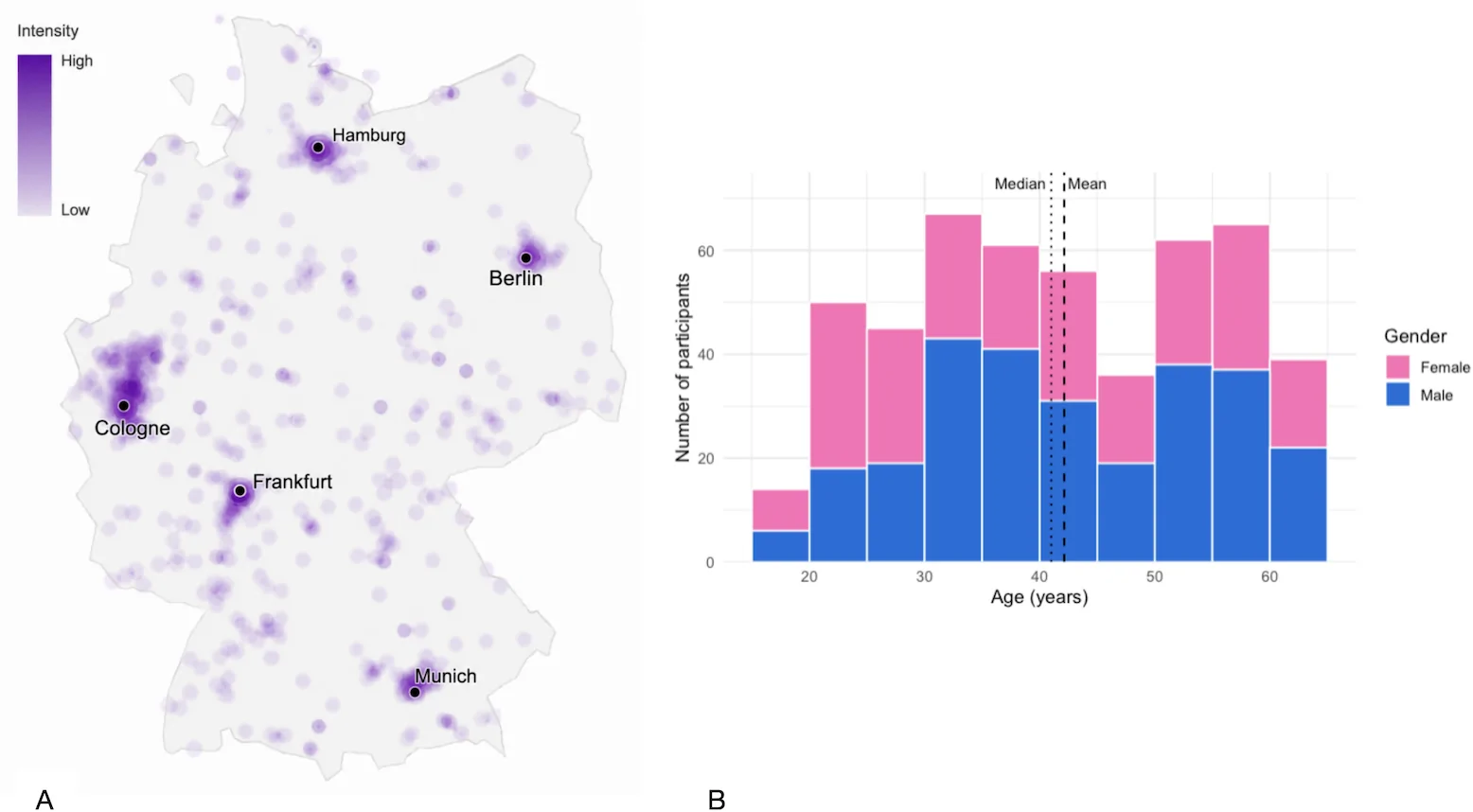
Characteristics of the study sample. (A) Geographic distribution of participants across Germany based on detected home locations from GPS data. The map includes a subsample of 477 participants with sufficient GPS data for home-location detection. Darker purple areas indicate greater overlap of detected home locations. Germany’s 5 largest cities are marked as geographic landmarks. (B) Age and gender distributions among participants with available demographic data (n=495). The bold dotted line indicates the mean age (42.12 years), and the thin dotted line indicates the median age (41.00 years).

Across 553 participants, we sampled a total of 7635 participant-days of sensing data. Although there is no consensus on a minimum sample size for multilevel latent profile models, sample size planning rules of thumb formulated by Park and Yu [63] recommend at least 20 Level 2 groups and 10 Level 1 units. From this sample, 495 participants provided their demographic information. Age ranged from 18 to 65 years, with an average of 42.12 (SD 12.89) years, and 44.65% (n=224) of participants indicated they were women, and 55.56% were men (n=220). Regarding education, approximately 35% had an intermediate secondary school qualification, 30% had a higher education entrance qualification, 20% had a university or higher education degree, and 14% had a lower secondary school qualification; only a small proportion reported having no school-leaving qualification or a doctorate or habilitation. Regarding employment status, 69% were employed, 10% were in education or training, 9% were not in the labor force, 7% had family or household responsibilities, and 5% were unemployed or seeking work, while only a very small proportion were engaged in voluntary or civic service. Regarding income, 20.6% reported €1501-€2000 (€1=US $1.1770 as of July 27, 2020), 18.6% reported €501-€1000, 17.6% reported €2001-€2500, 14.3% reported €1001-€1500, 6.7% reported €2501-€3000, 6.5% reported €3500 or more, 5.5% reported €3001-€3500, 5.3% reported less than €500, and 5.1% reported no income. See Figures 1A and 1B for an overview of the demographic characteristics of the sample.

### Sampling Procedures

Before recruitment began, quotas were established to approximate the German population in terms of age, gender, education, income, religious status, and relationship status [60]. These quotas guided the initial recruitment process. However, as recruitment progressed, some quota targets proved difficult to achieve, and the restrictions were subsequently relaxed to facilitate enrollment. Consequently, although recruitment was initially guided by the prespecified quota framework, the final sample did not strictly reflect the intended quota distribution.

### Sample Size, Power, and Precision

No a priori power analysis was performed because there is no established method for determining statistical power for multilevel LPA. Instead, we considered published recommendations for sample size in multilevel latent profile models. Although no consensus exists regarding the minimum required sample size, Park and Yu [63] recommended a minimum of 20 Level 2 units and 10 Level 1 units per Level 2 unit as a general guideline. In this study, Level 2 units corresponded to participants, and Level 1 units corresponded to daily observations. The final analytic sample included 553 participants and 7635 daily observations (mean 13.81, SD 0.83 days per participant), substantially exceeding these recommended minimum sample size guidelines.

### Study Design

This study used a 2-week intensive longitudinal observational design based on data from the SSPS. Participants installed the PhoneStudy app on their personal Android smartphones, which passively and continuously recorded smartphone use and sensor data during the study period. For the present analysis, sensing data from the 14 days preceding each participant’s completion of the mental well-being and personality questionnaires were examined. Daily observations were nested within participants, allowing behavioral patterns to be modeled at both the day and the person level. Specifically, multilevel LPA was used to identify recurring day-level behavioral profiles and higher-order person-level lifestyle profiles based on the distribution of these daily patterns. The resulting person-level profiles were then compared in terms of mental well-being, and Big Five personality traits were examined as potential moderators of the associations between lifestyle profiles and mental well-being. Because the study was observational, the analyses were intended to identify associations rather than establish causality.

### Ethical Considerations

The SSPS was reviewed and approved by the Ethics Committee of the Department of Psychology at LMU Munich under the study title “A Longitudinal Panel Study Combining Smartphone Sensing and Survey Methods.” At LMU Munich, the Institutional Review Board (IRB) does not assign approval numbers. Instead, ethical approval is granted and documented under the study title. Before participating, all individuals received detailed information about the study procedures, smartphone-sensing activities, data processing, compensation, and data protection measures in accordance with the General Data Protection Regulation. Participants provided informed consent before installing the PhoneStudy app and before beginning data collection. To protect privacy and confidentiality, participants were assigned randomly generated 8-character pseudonymous identifiers that were used to store and link sensing, experience-sampling, and survey data. Data were transferred to the study server through encrypted connections and analyzed using pseudonymized identifiers. Participants received monetary compensation for individual study activities, including monthly surveys, experience-sampling phases, and continuous smartphone sensing. Compensation increased with the duration of participation. Participants assigned to the 3-month study condition could receive up to €44.50 (€1=US $1.1770 as of July 27, 2020), whereas those assigned to the 6-month condition could receive up to €131.50 for completing all study activities. No images in the manuscript or supplementary materials permit identification of individual participants. Geographic information is presented only in an aggregated manner, and no individual GPS trajectories or precise residential locations are displayed.

### Measures and Covariates

#### Self-Report Measures

##### Mental Well-Being

The Warwick–Edinburgh Mental Well-Being Scale (WEMWBS) was used to assess mental well-being. It was developed by Tennant et al [3], and the German version was translated and validated by Lang and Bachinger [64]. Each item is rated on a 5-point Likert scale ranging from 1 (none of the time) to 5 (all of the time), with higher total scores indicating greater mental well-being. The WEMWBS was originally designed to capture multiple aspects of positive mental health, including positive affect (feelings of optimism, cheerfulness, and relaxation), positive functioning (energy, clear thinking, self-acceptance, personal development, competence, and autonomy), and satisfying interpersonal relationships [3]. Furthermore, previous psychometric research has identified both a strong general mental well-being factor and more specific domain factors. In German-speaking samples, bifactor models comprising a general well-being factor and 3 grouping factors representing positive affect, positive functioning, and interpersonal relationships have shown better fit than a strictly unidimensional model [64]. A 3-factor structure has also received support in German adult population samples [65]. We examined the factorial validity of the scale in this sample using confirmatory factor analysis. A correlated 3-factor model representing positive affect, positive functioning, and satisfying interpersonal relationships showed better fit than the one-factor model (see S3 in Multimedia Appendix 1). In the present study, participants rated every item on this scale based on their experiences over the past 2 weeks. The scale has been validated across various demographic and cultural groups and shows strong positive correlations with established measures of happiness, mental health, and life satisfaction, while negatively correlating with scales measuring anxiety and depression. The McDonald ω coefficients [66] of this scale were calculated.

##### Big Five Personality

The 15-item extra-short form of the Big Five Inventory–2 (BFI-2-XS) was used to assess personality. It was developed by Soto and John [67] and translated into a German version and validated by Rammstedt et al [68]. This inventory operationalizes personality structure by assessing the Big Five domains and 15 facets, including extraversion (with facets of sociability, assertiveness, and energy level), agreeableness (compassion, respectfulness, and trust), conscientiousness (organization, productiveness, and responsibility), negative emotionality (or so-called neuroticism; anxiety, depression, and emotional volatility), and open-mindedness (intellectual curiosity, aesthetic sensitivity, and creative imagination). In this inventory, each personality trait domain is covered with 3 items. The instrument uses a 1‐5 Likert scale, where 1 means strongly disagree and 5 means strongly agree. Although this inventory does not measure the specific facets of the traits, the short form effectively measures the Big Five personality domains [67,68].

### Smartphone Sensing Measures

The PhoneStudy app recorded many different types of smartphone-sensing data, providing information about participants’ daily lives. To capture the full richness of participants’ daily living patterns, we derived a set of digital phenotyping variables spanning 5 key domains known to be associated with mental well-being in synergy with one another—social engagement on smartphone, mobility, physical activity logs, physical sensor logs, and screen logs—by applying a suite of computational algorithms to raw sensor streams. Sensing features were computed for each person at the day level (12:00 AM-11:59 PM) according to pipelines described by Schoedel et al [69]. To improve LPA convergence and simplify interpretation, we selected 2 features for each of the 5 domains, prioritizing those with minimal missingness and low intercorrelations to avoid redundancy. A brief summary is shown in Table 1.

**Table 1.** Description of raw features extracted in this study. Citations for each category reflect research demonstrating that the domain synergistically relates to mental health in combination with the other listed behaviors or environments.

Category and feature		Description
Social engagement on smartphones [70]		
Communication app-use duration		Total duration of communication app usage
Social media app use duration		Total duration of social media app usage
Mobility [41]		
Travel distance		Total distance between consecutive GPS coordinates
Radius of gyration		Root-mean-square distance of place centroids
Physical activity [42]		
Walking probability		Average probabilities of walking
On-bicycle probability		Average probabilities of on-bicycle
Environment [43]		
Loudness		Average decibel level
Brightness		Average illuminance
Phone-use intensity [44]		
Total screen-on duration		Total screen-on time
Screen session count		Number of discrete screen-on events

### Social Engagement on Smartphones

We quantified social engagement by total time, in seconds, spent in communication apps and social media apps. App categories were derived from the systematic classification developed by Schoedel et al [71], which assigned 3091 commonly used Android apps to 26 psychologically meaningful categories. The classification system was developed iteratively and applied by 2 independent raters; disagreements were discussed and resolved with the involvement of a third rater. Interrater agreement was κ=.71 for communication apps and κ=.63 for social media apps. Communication apps were defined as apps whose primary purpose was direct interpersonal communication, including calling, text messaging, instant messaging, email, and video calling. Social media apps were defined as apps whose primary purpose was sharing, browsing, or interacting with content within an online community. Although many apps provide both functions, each app was assigned exclusively to one category according to its main functionality or primary selling point.

#### Mobility

Mobility was characterized using 3 GPS-derived features that captured daily travel extent, the number of stationary locations, and geographic dispersion.

- Total distance (m): sum of haversine distances between consecutive GPS coordinates, capturing daily travel extent [72].
- Number of clusters: number of stationary clusters identified via Density-Based Spatial Clustering of Applications with Noise (DBSCAN) [73]. The following parameters were used: *ε*=10  m (the maximum distance between 2 samples for one to be considered within the neighborhood of the other); min_samples=5 (the number of samples in a neighborhood for a point to be considered as a core point of a cluster).
- Radius of gyration (m): root-mean-square distance from each significant-place centroid to the participant’s overall centroid, quantifying geographic dispersion [74].

#### Physical Activity

Using the Android’s activity recognition snapshot API, providing transport-mode context beyond GPS alone [75], we computed the daily average probabilities of “walking,” “running,” and “on_bicycle” states—offering fine-grained separation of deliberate exercise from incidental movement.

#### Environment

Two ambient sensor features approximate participants’ acoustic and visual contexts: mean loudness (dB), defined as the average decibel level recorded via the microphone over each day; and mean brightness (lux-equivalent), defined as the average illuminance recorded by the ambient-light sensor.

#### Phone-Use Intensity

We quantified the intensity of phone usage patterns via total screen-on duration, defined as aggregate display-active time in seconds per day, and screen session count, defined as the number of discrete screen-on events, reflecting usage fragmentation and potential overstimulation.

### Data Collection

Data collection began in May 2020 and was conducted simultaneously for all participants. After enrollment, participants installed the self-developed Android-based PhoneStudy app on their personal smartphones, which continuously collected passive smartphone-sensing data in the background throughout the study period. Participants also completed monthly online questionnaires assessing demographic and psychological variables. For this study, only the survey assessing mental well-being and personality traits and the corresponding 14 days of smartphone-sensing data preceding survey completion were analyzed.

### Quality of Measurements

Smartphone-sensing variables were computed using previously validated processing pipelines. Daily features were derived automatically from raw sensor streams using standardized algorithms, minimizing observer-related measurement error. Self-report measures were administered online using validated German versions of established instruments.

### Instrumentation

Data were collected using the self-developed Android-based PhoneStudy app and online questionnaires. The PhoneStudy app continuously recorded passive smartphone-sensing data by accessing built-in Android system APIs and device sensors, including app use, GPS, activity recognition, ambient light, microphone, and screen-state information. Self-report data were collected through secure online questionnaires, including the WEMWBS and the BFI-2-XS, both of which are validated German-language instruments. Smartphone-sensing features were derived from the raw sensor streams using previously validated processing pipelines described by Schoedel et al [69].

### Masking

Because this was an observational study without experimental conditions, masking or blinding of participants, investigators, or outcome assessors was not applicable.

### Psychometrics

For the German version of the WEMWBS, because the present study examined both the composite score and 3 subdimensions, we first evaluated the factorial validity of the instrument using confirmatory factor analysis. A correlated 3-factor model representing positive affect, positive functioning, and satisfying interpersonal relationships provided a better fit than a one-factor model (see S3 in Multimedia Appendix 1). Internal consistency in the current sample was excellent for the composite score (*ω*=.95), positive affect (*ω*=.95), positive functioning (*ω*=.84), and satisfying interpersonal relationships (*ω*=.83).

For the German version of the BFI-2-XS, previous validation studies reported Cronbach α coefficients ranging from 0.45 to 0.67, reflecting the brevity of the 3-item scales, and test-retest reliabilities ranging from 0.72 to 0.88 [68]. In the present sample, McDonald ω coefficients were 0.44 for extraversion, 0.58 for agreeableness, 0.55 for conscientiousness, 0.75 for negative emotionality, and 0.50 for openness. These reliability estimates are comparable to those reported in the original validation study and are consistent with expectations for very brief personality measures.

### Conditions and Design

No experimental manipulation was implemented. The study used a nonexperimental, observational, intensive longitudinal design with repeated daily observations nested within participants.

### Data Diagnostics

We conducted an attrition analysis to examine whether participants retained in the analysis differed from those excluded between enrollment and analysis. No significant differences were found in age, gender, education, income, religion, or relationship status; full results are reported in S1 in Multimedia Appendix 1. We further compared the demographic composition of the final sample with the initial quota design using chi-square goodness-of-fit tests. The final sample retained the initial quota composition for age, gender, and relationship status, whereas significant deviations were observed for education, income, and religious status. See S2 in Multimedia Appendix 1 for details.

### Analytic Strategies

#### Feature Preprocessing

Feature preprocessing was performed to ensure that the sensing variables met the distributional assumptions of subsequent latent profile analyses, which require approximate within‐class normality [50]. Some of the original features exhibited pronounced skew (|skewness|>1), risking biased parameter estimates and poor model fit. To address this, we applied Box-Cox power transformations [76], a likelihood‐based method that identifies an optimal exponent (λ) to stabilize variance and render distributions more symmetric [77]. This approach attenuated the influence of extreme values, improved the efficiency of parameter estimation, and enhanced convergence in mixture modeling frameworks [78]. After transformation, all previously skewed features closely approximated normal distributions, thereby bolstering the robustness and interpretability of our multivariate analyses.

#### Missing Data Handling

Applying the 4/7 validity rule [62] ensured that each participant contributed ≥8 valid days within the 14-day window. For participants with fewer than 14 valid days (n=39), we did not impute values of all features for the missing days because day-level profile proportions could be computed from observed days in 2-level LPA. Missing values within sensing features were assumed missing at random (MAR), primarily due to temporary device/app malfunctions rather than participant choice. Therefore, they were handled with a full information maximum likelihood (FIML) estimation [79], which is the default function to deal with the missing data in Mplus (version 8.3; Muthén & Muthén), given the missing values are at random.

#### Latent Profile Analyses

##### Overview

Ten sensing features’ values were standardized and used as inputs for the LPA in Mplus version 8.3. To reduce the likelihood of convergence on local solutions, latent profiles were estimated with 1000 random start values [51,52]. We specified up to 10 latent profiles in which the means of all variables were freely estimated across all profiles. To avoid the potential problem of overparameterization, which may cause improper or nonconvergent solutions, the variances of all variables across profiles were fixed [80-82].

To identify the optimal model with an adequate number of latent profiles/classes at both levels, we followed the 3-step procedure recommended by Lukočienė et al [83]. First, we conducted a series of LPAs (ignoring the multilevel structure) to determine the optimal number of profiles at Level 1 (ie, profiles of lifestyles at the daily level). Second, we specified multilevel-latent profile analysis (ML-LPA) models and fixed the number of Level 1 profiles to the best solution of the first step and determined the number of Level 2 (person-level) classes. Third, we re-evaluated the number of Level 1 (day-level) profiles by fixing the number of person-level classes to the best solution from the second step. The aim of the third step was to evaluate whether the number of Level 1 profiles changed after accounting for the multilevel data structure.

To find the best-fitting model and describe the quality of profile separation, we considered the following criteria: (1) Bayesian information criterion (BIC [84]) and the Akaike information criterion (AIC [85]). Lower values indicate better model fit [86,87]. The elbow rule was applied, identifying the point after which the additional profiles yielded only marginal improvements relative to the increase in model complexity [53]. (2) Entropy, which measures the ability of a mixture model to provide well-separated clusters [88]. Entropy can range from 0 to 1, with higher values representing a better fit of the profiles to the data [89]. Furthermore, the theoretical coherence and interpretability of the selected profile solution need to be taken into consideration when determining the number of profiles that are retained [90].

##### Differences in Mental Well-Being Across Profiles

For the final model, we analyzed whether the person-level profiles differed in mean levels of mental well-being, including the composite score and 3 subdimension scores. For the mean comparisons across person-level profiles, we applied the adjusted 3-step approach [91] by R (version 4.4.1; R Foundation for Statistical Computing) [92]. This is a misclassification-correction approach for estimating profile-specific means of an outcome. Individual posterior profile-membership probabilities were first obtained and then converted into an assigned class indicator. Using these posterior probabilities and assignment weights, we estimated the misclassification matrix Q, where ${\text{Q}}_{\text{r}\text{,}\text{t}}\text{=}\text{P}\text{(}\text{X}\text{=}\text{t}\text{\textbackslash{}mid }\text{W}\text{=}\text{r}\text{)}$ links the assigned profile W to the true latent profile X. Naive outcome means by assigned profile${\text{m}}_{\text{naive}}\text{=}\text{E}\text{[}\text{Z}\text{∣}\text{W}\text{]}$, were then adjusted by solving the linear mixture relationship ${\text{m}}_{\text{naive}}\text{=}\text{Qμ}$, yielding adjusted latent-profile means$\text{μ}\text{=}{\text{Q}}^{\text{−1}}{\text{m}}_{\text{naive}}$. The statistical significance of the mean differences in mental well-being scores across classes was evaluated using omnibus Wald tests [93]. For any mental well-being dimension that showed significant differences across profiles, we subsequently conducted pairwise comparisons to identify differences between person-level profiles.

##### Moderating Effects of Big Five Personality Traits

Moderation was tested using multiple linear regression models in which well-being was regressed on personality traits and profile membership indicators. Personality was represented by the Big Five trait scores. Profile membership was represented by posterior probabilities of belonging to each latent profile/class derived from the latent profile model. One profile probability was omitted as the reference category to avoid perfect multicollinearity (ie, probabilities across profiles sum to 1).

To test whether the association between profile membership and well-being differed as a function of personality, we fit hierarchical regression models. In the baseline model, well-being was predicted from the 5 personality traits and the 6 profile-probability predictors:


$$[WB=\beta_{0}+\sum_{i=1}^{5}\beta_{i}({\text{Trait}}_{i})+\sum_{j=1}^{6}\gamma_{j}({\text{prob}}_{BC_{j}})+\varepsilon$$

In the moderation model, we added the full set of cross-product interaction terms between each personality trait and each profile-probability predictor (5×6=30 interaction terms):


$$WB=\beta_{0}+\sum_{i=1}^{5}\beta_{i}({Trait}_{i})+\sum_{j=1}^{6}\gamma_{j}({prob}_{BCj})+\sum_{i=1}^{5}\sum_{j=1}^{6}\delta_{ij}({Trait}_{i}\times {prob}_{BCj})+\varepsilon$$

Moderation was evaluated via the incremental variance explained by adding the interaction block, quantified as Δ*R*², and tested using an omnibus *F* test comparing the moderation model to the baseline model (ie, testing the null hypothesis that all interaction coefficients ${\text{δ}}_{\text{ij}}$equal 0). Statistical significance was evaluated using a 2-sided *α*=.05.

## Results

### Participant Flow

**Figure 2.**
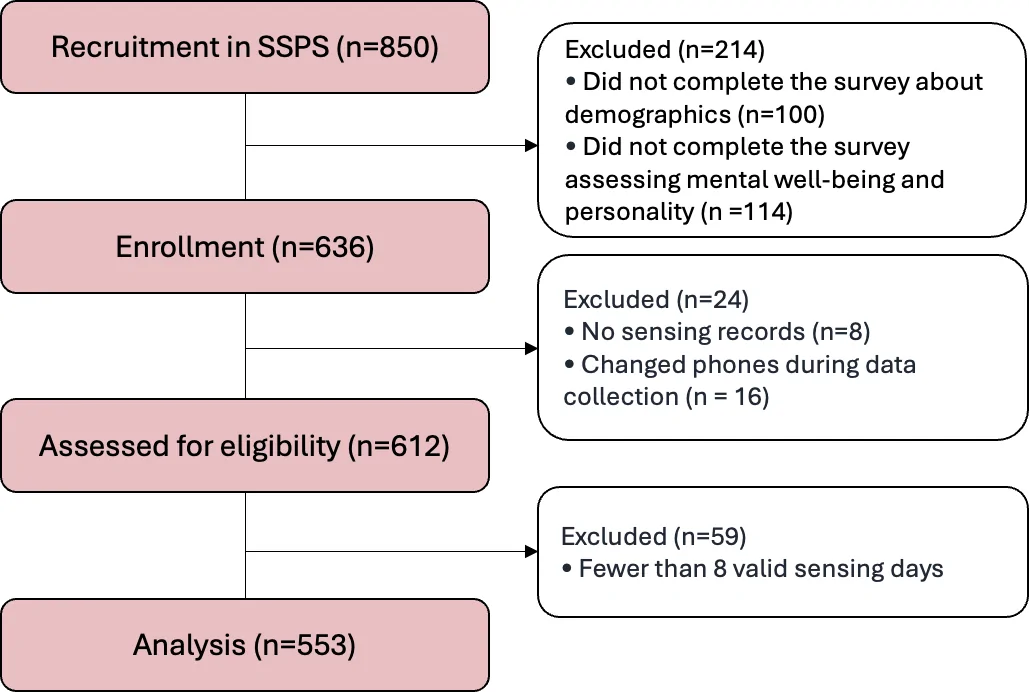
Flowchart of participants according to CONSORT (Consolidated Standards of Reporting Trials) guidelines [94]. Full details of participant recruitment for the Smartphone Sensing Panel Study (SSPS) are provided in the study protocol [60]. SSPS: Smartphone Sensing Panel Study.

The flow of participants from recruitment to the final analytic sample is presented in Figure 2.

### Recruitment

Participant recruitment was conducted between May and November 2020 as part of the SSPS. The questionnaire wave analyzed in the present study was completed in July 2020, and smartphone-sensing data from the preceding 14 days were included in the analyses.

### Descriptive and Correlation Analyses

For the current study, 10 features spanning five domains were computed per participant and day: (1) social engagement on smartphones: communication app usage duration, social media app usage duration, (2) mobility: travel distance, radius of gyration, (3) physical activity: walking probability, on-bicycle probability, (4) environment: loudness and brightness, (5) phone-use intensity: screen-on duration and screen session count. Of these 10 features, 8 exhibited high skewness and thus were Box-Cox transformed (see Figure S1 in Multimedia Appendix 1 for details). Subsequently, all variables were *z* score normalized to obtain standardized values across variable types. See Table 2 for correlational results.

**Table 2.** Descriptive statistics and correlations for sensing variables and mental well-being (N=553). Correlations were calculated by aggregating the sensing data to the personal level. All *P* values were adjusted with Bonferroni correction across all pairwise correlations.

Variable	Mental well-being	Positive affect	Positive functioning	Personal relationship	Communication app-use duration	Social media app-use duration	Travel distance	Radius of gyration	Walking probability	On-bicycle probability	Loudness	Brightness	Screen-on duration	Screen session count
Mental well-being	—^a^	.93^b^	.86^b^	.87^b^	–.06	–.06	.10	.08	.08	.04	–.06	.03	–.21^b^	–.01
Positive affect	.93^b^	—	.70^b^	.71^b^	–.06	–.04	.12	.09	.08	.06	–.06	.03	–.18^b^	.02
Positive functioning	.86^b^	.70^b^	—	.66^b^	–.13	–.13	–.01	.01	.03	–.02	–.07	–.01	−.24^b^	–.08
Personal relationship	.87^b^	.71^b^	.66^b^	—	.02	–.01	.13	.10	.10	.05	–.04	.07	–.15^b^	.01
Communication app-use duration	–.06	–.06	–.13	.02	—	.31^b^	.13	.11	.14	.17^b^	–.06	.18^b^	.48	.45^b^
Social media app-use duration	–.06	–.04	–.13	–.01	.31^b^	—	.10	.09	.11	.09	.02	.15	.39^b^	.33^b^
Travel distance	.10	.12	–.01	.13	.13	.10	—	.79^b^	.40^b^	.45^b^	.21^b^	.33^b^	.02	.30^b^
Radius of gyration	.08	.09	.01	.10	.11	.09	.79^b^	—	.39^b^	.48^b^	.15	.22^b^	–.03	.25^b^
Walking probability	.08	.08	.03	.10	.14	.11	.40^b^	.39^b^	—	.55^b^	.18^b^	.34^b^	.03	.42^b^
On-bicycle probability	.04	.06	–.02	.05	.17^b^	.09	.45^b^	.48^b^	.55^b^	—	.27^b^	.30^b^	.04	.39^b^
Loudness	–.06	–.06	–.07	–.04	–.06	.02	.21^b^	.15	.18^b^	.27^b^	—	.40*	–.11	.33^b^
Brightness	.03	.03	–.01	.07	.18^b^	.15	.33^b^	.22^b^	.34^b^	.30^b^	.40^b^	—	.01	.52^b^
Screen-on duration	–.21^b^	–.18^b^	–.24^b^	–.15^b^	.48	.39^b^	.02	–.03	.03	.04	–.11	.01	—	.26^b^
Screen session count	–.01	.02	–.08	.01	.45^b^	.33^b^	.30^b^	.25^b^	.42^b^	.39^b^	.33^b^	.52^b^	.26^b^	—

a Not applicable.

b *P*<.05.

### Statistics and Data Analysis

#### Missingness Test

Cluster-adjusted missingness-indicator analyses showed that missingness in travel distance and radius of gyration was associated with observed within-person variables, whereas no such associations were detected for other variables (see S4 in Multimedia Appendix 1). Multiple imputation was considered but not implemented because a defensible imputation model would have needed to preserve the multilevel structure, mixed and skewed indicator distributions, relationships among derived sensing features, and compatibility with the multilevel latent profile model. The analyses were therefore estimated in Mplus using robust maximum likelihood (MLR) under an MAR assumption, conditional on the observed information included in the model.

#### Identification of Profiles

To determine the best solution for the multilevel LPA, we followed a 3-step procedure [83]. First, to identify day-level profiles, we fixed the number of Level-2 profiles at 1 and varied the number of Level-1 profiles from 1 to 10 to find the optimal Level-1 solution (see Table 3). Lower BIC and AIC values indicate better model fit. However, these indices may continue to decrease as additional latent classes are extracted. In such cases, the optimal solution is typically identified at the point where further decreases begin to level off. We chose the 8-profile solution because both AIC and BIC showed the largest decrease when moving from 7 to 8 profiles, whereas additional profiles (9-10) yielded only minor further improvements. Additionally, entropy showed little improvement from the 7-profile to the 8-profile solution and was lower for solutions with more than 8 profiles, supporting the 8-profile solution as a well-separated and parsimonious classification. While some researchers argued that profiles should not contain less than 5% of the sample, the most important consideration when deciding if a profile size is too small is whether the solution is supported by model fit statistics and whether the small profile makes conceptual sense. Given the large day-level sample (n=7635) and the fact that the smallest profile captures a meaningful subgroup (see below), we did not apply a hard 5% threshold for class size. Second, to identify person-level profiles, we fixed Level-1 to the 8-profile solution and varied the number of Level-2 profiles from 1 to 10 to find the best Level-2 solution. Based on the AIC and BIC, we selected the 7-profile solution because additional profiles provided only marginal improvements. Entropy also remained high for 7 profiles, confirming this as a quite parsimonious solution. Finally, we re-examined the Level-1 profiles by fixing Level-2 to the 7-profile solution, re-estimating models with 1 to 10 Level-1 profiles. This time, the fit indices exhibited the largest improvement when moving from 7 to 8 profiles. Consequently, we selected a model with 8 profiles at Level-1 and 7 profiles at Level-2.

**Table 3.** Model fit statistics for latent profile analysis (LPA) models step 1‐3.

Class (level 1)	Class (level 2)	BIC^a^	Sample size-adjusted BIC	△BIC	LL^b^	AIC^c^	△AIC	*N*par	Entropy (level 1)	Size of smallest profile (level 1)	Size of smallest profile (level 2)
Step1: model fit statistics for LPA^d^ models with different numbers of Level 1 profiles (and 1 level 2 profile)											
1	1	207234.509	207170.954	—	–103527.85	207095.699	—	20	1	1	—
2	1	196912.443	196813.931	10322.066	–108705.326	196697.287	10398.412	31	0.957	0.233	—
3	1	191977.646	191844.178	4934.797	–95801.072	191686.145	5011.142	42	0.948	0.158	—
4	1	187544.41	187375.987	4433.236	–103910.989	187176.563	4509.582	53	0.967	0.079	—
5	1	185330.383	185127.005	2214.027	–92379.096	184886.191	2290.372	64	0.965	0.028	—
6	1	183186.632	182948.298	2143.751	–91258.048	182666.095	2220.096	75	0.972	0.012	—
7	1	181651.191	181377.901	1535.441	–90441.154	181054.308	1611.787	86	0.921	0.012	—
8	1	179624.157	179315.911	2027.034	–89378.464	178950.929	2103.379	97	0.921	0.012	—
9	1	178422.983	178079.781	1201.174	–88728.704	177673.409	1277.52	108	0.914	0.012	—
10	1	177525.255	177147.098	897.728	–88230.668	176699.336	974.073	119	0.896	0.012	—
Step 2: model fit statistics for ML-LPA^e^ models with different numbers of level 2 profiles (and 8 level 1 profiles)											
8	1	179624.157	179315.911	—	–89378.464	178950.929	—	97	0.921	—	1
8	2	174435.67	174102.001	5188.487	–86748.459	173706.917	5244.012	105	0.932	—	0.466
8	3	172634.256	172275.166	1801.414	–85811.99	171849.98	1856.937	113	0.952	—	0.288
8	4	171634.298	171249.785	999.958	–85276.249	170794.498	1055.482	121	0.955	—	0.159
8	5	170901.255	170491.320	733.043	–95149.382	170005.931	788.567	129	0.952	—	0.087
8	6	170455.686	170020.328	445.569	–84615.419	169504.837	501.094	137	0.955	—	0.040
8	7	170096.119	169635.339	359.567	–94662.441	169089.747	415.09	145	0.955	—	0.040
8	8	169937.376	169451.174	158.743	–84284.74	168875.48	214.267	153	0.954	—	0.040
8	9	169857.042	169345.417	80.334	–94488.084	168739.621	135.859	161	0.956	—	0.018
8	10	169836.679	169299.632	20.363	–84162.867	168663.735	75.886	169	0.956	—	0.018
Step 3: model fit statistics for ML-LPA models with different numbers of level 1 profiles (and 7 level 2 profiles)											
1	7	207288.152	207205.530	—	–103527.85	207107.699	—	26	0.615	1	—
2	7	191976.897	191840.252	15311.255	–95796.228	191678.456	15429.243	43	0.796	0.442	—
3	7	185115.851	184925.184	6861.046	–92289.711	184699.422	6979.034	60	0.909	0.174	—
4	7	180158.948	179914.258	4956.903	–89735.265	179624.53	5074.892	77	0.949	0.080	—
5	7	177203.091	176904.378	2955.857	–88181.342	176550.684	3073.846	94	0.954	0.029	—
6	7	174593.325	174240.590	2609.766	–86800.465	173822.93	2727.754	111	0.954	0.028	—
7	7	172499.884	172093.126	2093.441	–85677.75	171611.5	2211.43	128	0.955	0.013	—
8	7	170096.119	169635.339	2403.765	–94662.441	169089.747	2521.753	145	0.955	0.013	—
9	7	168830.08	168315.278	1266.039	–83690.86	167705.719	1384.028	162	0.956	0.013	—
10	7	167770.201	167201.376	1059.879	–83084.926	166527.852	1177.867	179	0.941	0.013	—

a BIC: Bayesian information criterion.

b LL: log-likelihood.

c AIC: Akaike information criterion,

d LPA: latent profile analysis.

e ML-LPA: multilevel-latent profile analysis.

#### Interpretation of Day-Level Profiles

To enhance profile interpretability, we computed the means of all variables for each profile (see Table 4). The raw distributions of all 10 indicators before transformation and standardization can be found in Multimedia Appendix 1. Profile 1 summarized days with much social media app time, extensive mobility and physical activity, loud and bright environments, frequent phone sessions, and long screen time. Profile 1 days could therefore be interpreted as days with a wide range of movement, frequent phone checking, and heavy social media use, for example, commuting or long-distance travel days when people use their smartphones on the way.

**Table 4.** Latent profiles of sensing variables on day-level. The results are drawn from the 7 person-level and 8 day-level profile solution.

Profile	Communication^a^, mean (SD)	Social media^b^, mean (SD)	Distance, mean (SD)	Gyratio, mean (SD)	Walking, mean (SD)	On-bicycle, mean (SD)	Loudness, mean (SD)	Brightness, mean (SD)	Screen count, mean (SD)	Screen time, mean (SD)	Days (n)	Probability
1	0.362 (0.837)	1.248 (0.234)	0.253 (0.841)	0.223 (0.940)	0.415 (0.699)	0.351 (0.735)	0.19 (0.683)	0.282 (0.830)	0.57 (0.813)	0.385 (0.647)	2012	0.264
2	–0.574 (1.048)	–0.867 (0.188)	–1.054 (0.800)	–0.944 (0.620)	–1.426 (0.132)	–1.314 (0.327)	–0.078 (0.801)	–0.422 (0.919)	–0.822 (0.888)	–0.626 (1.208)	1018	0.133
3	–0.06 (0.974)	1.122 (0.370)	–0.911 (0.717)	–0.802 (0.656)	–1.395 (0.245)	–1.284 (0.408)	–0.017 (0.746)	–0.197 (0.843)	–0.192 (0.871)	0.352 (0.869)	518	0.068
4	–0.674 (0.822)	–0.888 (0.133)	0.007 (0.850)	–0.016 (0.962)	0.513 (0.791)	0.43 (0.862)	–0.119 (0.748)	–0.407 (0.947)	–0.636 (0.681)	–0.696 (0.926)	1191	0.156
5	0.251 (0.845)	–0.873 (0.143)	0.421 (0.870)	0.369 (0.921)	0.374 (0.709)	0.371 (0.755)	0.356 (0.706)	0.453 (0.825)	0.398 (0.796)	0.108 (0.741)	1760	0.231
6	1.487 (0.962)	1.322 (0.416)	–0.533 (1.344)	–0.609 (0.950)	–1.069 (0.849)	–1.076 (0.760)	–3.827 (0.466)	–2.162 (0.254)	–1.743 (0.694)	2.257 (0.819)	97	0.013
7	0.095 (0.872)	0.41 (0.308)	0.331 (0.874)	0.345 (0.894)	0.312 (0.742)	0.372 (0.826)	0.143 (0.630)	0.193 (0.848)	0.249 (0.756)	–0.002 (0.760)	905	0.119
8	0.142 (1.515)	–0.839 (0.255)	–1.046 (1.159)	–0.856 (0.793)	–0.809 (1.013)	–0.739 (1.099)	–3.884 (0.198)	–2.176 (0.133)	–1.583 (0.735)	0.762 (1.762)	134	0.018

a Communication: communication apps usage duration.

b Social media: Social media app usage duration.

Profile 2 subsumed days with little phone usage and low movement. Days in this profile could therefore be interpreted as low-engagement days during which individuals spent little time using or near their phones.

Profile 3 represents days with high social media app use and average communication app use. Both mobility and physical activity are similar to profile 2. The screen time was long, while the screen frequency is below average. Such days may be interpreted as sedentary days characterized by social media use.

In profile 5, a typical day exhibited active movement and mobility like in profile 1, but with much less social media app usage and higher loudness and brightness levels. These days also showed above-average app usage and screen frequency with near-average screen time. Profile 5 could therefore be interpreted as commuting or travel days, just like profile 1, while the phone is used in short bursts and not for social apps and in relatively stimulating or exposed environments.

Profile 6 represented days with very long total screen time across only a few sessions in tranquil and dark settings. This profile could represent deep-use days—binging on the phone indoors.

Profile 7 shows days with moderate communication and social media app usage, high loudness and brightness, and approximately average screen time. Its mobility and physical activity levels were similar to profile 5, but both phone-use frequency and duration were lower, and these days occurred in relatively noisy and bright environments. These patterns suggest days spent commuting or moving, with moderate levels of phone and social media checking.

Profile 8 represented days with mobility, physical activity, and an environment similar to profile 6, but with lower (but still high) screen time and social app usage. These may be days when users were indoors with sustained screen use, although not primarily driven by social media apps.

#### Interpretation of Person-Level Profiles

**Figure 3.**
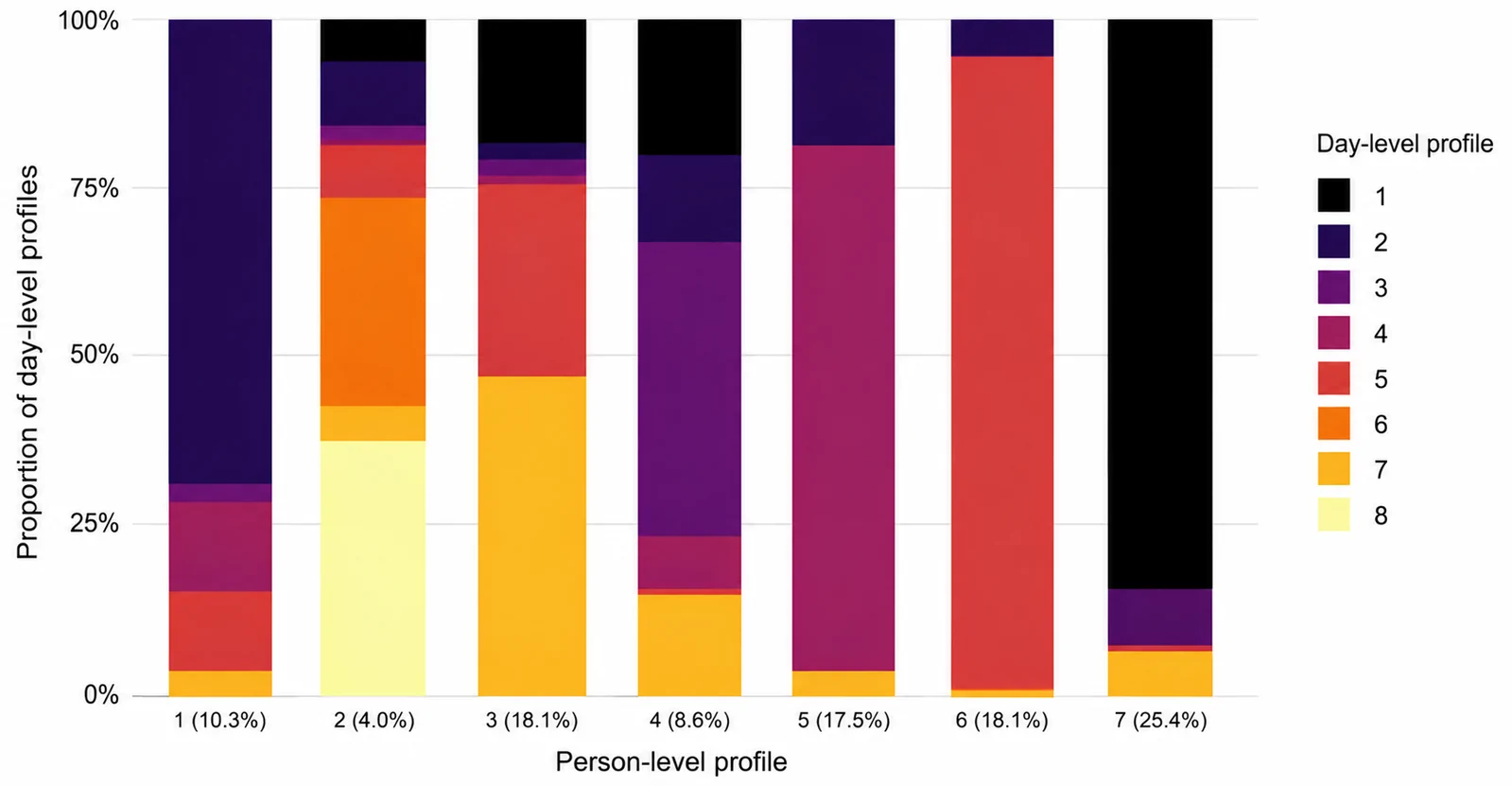
Profiles of proportions of daily levels on the personal level. Distribution of day-level behavioral profiles across person-level profiles in a 14-day intensive longitudinal smartphone-sensing study of 553 adults in Germany, July 2020. The figure shows the estimated proportion of days assigned to each day-level profile within each person-level profile identified through multilevel latent profile analysis.

Figure 3 displays the final ML-LPA model with 8 profiles at Level 1 and 7 profiles at Level 2.

In Profile 1 (n=57), people spent roughly two-thirds of their days with minimal engagement (day-level profile 2) and, on their remaining days, exercised (day-level profile 4) or commuted (day-level profile 5). The proportions were calculated using a hard assignment, whereby each participant was assigned to the profile with the highest posterior probability. However, LPA yields a full set of posterior probabilities for each participant across all profiles, rather than a single definitive label. This profile could represent individuals who spent most days away from their phones, with occasional physical activity and phone usage.

Profile 2 (n=26) comprised people who frequently showed day-level patterns of sustained screen use with apps other than social media (day-level profile 8) and deep social media engagement (day-level profile 6). This represented a small group concentrated in “indoor” days with intensive phone use.

In Profile 3 (n=88), nearly half of the days were spent moving while checking phones at a medium level (day-level profile 7). The other half was spent in a commute-like manner, either with little social media use (day-level profile 5) or many phone checks and strong social media use (day-level profile 1). People in this profile could be long-distance commuters or travelers, with varied phone and social app usage.

In profile 4 (n=48), people spent most of their days sedentary with intensive social media use (day-level profile 3). However, days in this profile were not dominated by a single routine but were spread across day-level profiles 1, 2, 4, and 7. Social media apps were used on most days, although people in this profile also had days characterized by mobility and smartphone use.

People in profile 5 (n=96) spent more than 70% of their days engaged in exercise and low digital activity (day-level profile 4). The remaining days were characterized by low phone engagement (day-level profile 2). These people typically spent much of their time walking or cycling and were largely unplugged from smartphone or social media app use.

In profile 6 (n=99), the dominant days were commute-type days (day-level profile 5), characterized by above-average mobility and physical activity, low social-media use, frequent but relatively brief phone interactions, and comparatively loud and bright environments. They were accompanied by some low-engagement days (day-level profile 2). These could be people on the go with frequent phone checks, but not for communication or social media use.

In profile 7 (n=139), almost 80% of days were busy days with lots of phone checks and strong social media app use (day-level profile 1), with high social media use, frequent phone sessions, above-average mobility and physical activity, and high loudness and brightness. These days were accompanied by some sedentary days characterized by social media use (day-level profile 3). People in this profile exhibited frequent phone checks and social media use while being mobile, with occasional stationary social days.

To demonstrate the added value of the 2-level LPA, we conducted a supplementary single-level LPA using person-level averages of the sensing variables across the 2-week observation period. The single-level solution primarily captured overall behavioral intensity and obscured differences in the composition and recurrence of distinct daily patterns, supporting the use of the 2-level approach (see S5 in Multimedia Appendix 1).

#### Differences Between Personal Profiles in Mental Well-Being

We analyzed whether the mean values of the well-being measures differed across person-level profiles. Means and SDs of the adjusted average scores of mental well-being and its 3 subscales across the different profiles of individuals are displayed in Table 5. The profiles identified are not hard labels; rather, each case has one probability for each profile. Adjusted mean refers to the mean value accounting for classification error in profile assignment.

**Table 5.** Adjusted means of well-being outcomes across person-level profiles. Adjusted mean refers to the mean value accounting for classification error in profile assignment.

Person-level profile	Mental well-being, mean (SD^a^)	95% CI	Positive affect, mean (SD^a^)	95% CI	Positive functioning, mean (SD^a^; 95% CI)	95% CI	Personal relationship, mean (SD^a^; 95% CI)	95% CI
1: Indoor and low engagement	3.41 (0.78)	(3.20-3.63)	3.16 (0.86)	(2.92-3.39)	3.74 (0.85)	(3.51-3.98)	3.46 (0.83)	(3.24-3.69)
2: Indoor and deep use)	3.60 (0.66)	(3.35-3.85)	3.45 (0.80)	(3.15-3.75)	3.81 (0.78)	(3.52-4.10)	3.63 (0.68)	(3.37-3.88)
3: Mobile and mixed level social	3.54 (0.63)	(3.40-3.67)	3.37 (0.69)	(3.22-3.52)	3.76 (0.66)	(3.62-3.90)	3.57 (0.74)	(3.41-3.73)
4: Social-heavy and sedentary-lean	3.47 (0.72)	(3.26-3.68)	3.22 (0.81)	(2.99-3.46)	3.84 (0.75)	(3.62-4.06)	3.47 (0.81)	(3.23-3.71)
5: Physically active and unplugged	3.54 (0.63)	(3.41-3.67)	3.33 (0.78)	(3.17-3.49)	3.94 (0.63)	(3.81-4.06)	3.48 (0.73)	(3.33-3.63)
6: Commute-style and bursty use	3.55 (0.63)	(3.42-3.67)	3.38 (0.71)	(3.24-3.52)	3.79 (0.61)	(3.66-3.91)	3.56 (0.80)	(3.40-3.72)
7: Mobile and always-on social	3.45 (0.69)	(3.33-3.56)	3.28 (0.80)	(3.14-3.41)	3.61 (0.74)	(3.48-3.73)	3.55 (0.73)	(3.43-3.67)

a SDs were calculated from the raw observed values within each profile, whereas the reported means and 95% CIs were adjusted for classification error.

We computed Wald tests to see if any of the 7 person-level profiles differed significantly in well-being. As shown in Table 5, only the result for positive functioning was significant, showing that at least some of the personal profiles differed in their adjusted mean positive functioning (*χ*^2^_6_=13.39; *P*=.04). See table S9 in Multimedia Appendix 1 for results of pairwise comparisons.

Across the 7 person‐level profiles, average positive functioning ranged from 3.61 to 3.94 (on a scale from 1 to 5). Individuals assigned to person-level profile 7 reported a lower level of positive functioning (mean 3.61, SD 0.74; 95% CI 3.48-3.73) compared with profile 5 (mean 3.94, SD 0.63; 95% CI 3.81-4.06; Cohen *d*=0.47; 95% CI 0.21-0.73), suggesting that people who spent their days in extended mobility and sustained social-media engagement showed lower levels of positive functioning. Person-level profile 5, characterized by greater proportions of physically active, low-digital-engagement days (eg, those dominated by day-level profile 4), exhibited the highest level of positive functioning. Only these 2 extreme profiles differed significantly. The other 5 person-level profiles exhibited no significant difference with either profile 7 or profile 5 after Holm adjustment [95]. Together, these results suggest that multilevel latent profiles of sensing features could capture certain variation in positive functioning, an important aspect of mental well-being. A sensitivity analysis excluding the smallest person-level profile, profile 2, yielded the same results. Specifically, positive functioning remained the only well-being outcome that differed significantly across profiles, supporting the robustness of the original findings. See S6 in Multimedia Appendix 1 for details.

We additionally examined whether the 7 person-level profiles differed across demographic characteristics, including age, gender, relationship status, educational level, and income. Only age showed a robust difference across profiles, Wald *χ*²_6_=78.62; *P* <.001, with the mobile and always-on social profile (profile 7) being the youngest on average (mean age 35.38, SD 11.43). No significant profile differences were found for gender, relationship status, or income. Full results are reported in S7 in Multimedia Appendix 1.

#### Interactions of Profiles and Personalities in Well-Being Prediction

Table 6 presents the interaction effects of profile×personality predicting 4 well-being outcomes. Across all outcomes, adding the full set of profile×personality interaction terms did not significantly improve model fit relative to the main-effects models (all Δ*F* tests ns), indicating no omnibus evidence that personality moderated the associations between profile membership and well-being.

**Table 6.** Hierarchical regression models testing personality moderation of profile-probability–well-being associations (n=553). The baseline model included extraversion, openness, conscientiousness, agreeableness, and negative emotionality (neuroticism) and probabilities of profile 1-6. Moderation model added all trait×profile-probability interactions (5×6=30 terms). Δ*F* tests the increment in explained variance from adding the interaction block. In these models, profile 7 was treated as the reference profile by omitting its posterior probability.

Outcome	Baseline model		Moderation model		Comparison		
	*R*²	*F* test (*df*)	*R*²	*F* test (*df*)	Δ*R*²	Δ*F* test (*df*)	*P* value (ΔF)
Mental well-being	0.50	49.76 (11, 541)	0.53	13.98 (41, 511)	0.03	0.93 (30, 511)	.57
Positive affect	0.39	31.99 (11, 541)	0.43	9.213 (41, 511)	0.03	0.92 (30, 511)	.60
Positive functioning	0.50	49.21 (11, 541)	0.53	14.00 (41, 511)	0.03	1.04 (30, 511)	.41
Personal relationship	0.34	25.27 (11, 541)	0.37	7.197 (41, 511)	0.03	0.72 (30, 511)	.87

Notably, all 4 omnibus tests were nonsignificant, with *P* values ranging from .41 to .87. Therefore, the conclusion would remain unchanged under a Bonferroni correction for 4 outcomes or any less conservative correction.

## Discussion

### Principal Findings

To our knowledge, this is the first study to use a latent profile framework with mobile sensing data to characterize daily behavioral patterns for digital phenotyping. By capturing smartphone-based records of social engagement, mobility, physical activity, environment, and phone-use intensity, we identified 8 daily behavioral profiles and aggregated them into 7 person‐level profiles. In this German adult sample, we found that person-level profiles did not differ in composite mental well-being or 2 of its subscales (ie, positive affect and satisfying interpersonal relationships), but did differ in a third subscale, positive functioning. Specifically, a profile characterized by high physical activity and low digital engagement showed higher levels of positive functioning compared to the profile marked by extensive mobile and social media use. Moreover, we found no evidence that personality moderated these associations in the current sample. The findings primarily demonstrated the feasibility of deriving interpretable multilevel behavioral profiles from mobile-sensing data, whereas evidence that these profiles meaningfully distinguish mental well-being was limited and requires replication.

### Lifestyle Profiles and Mental Well‐Being

The overall pattern of findings indicates that the identified lifestyle profiles were only weakly and selectively associated with mental well-being. Across the 7 person-level profiles, we observed no significant differences in the composite mental well-being score, positive affect, or satisfying interpersonal relationships. Moreover, most pairwise comparisons of positive functioning were also nonsignificant. The only significant contrast indicated higher positive functioning in the “active and unplugged” profile than in the “mobile and always-on social” profile. Accordingly, the results do not support that the profile solution consistently distinguishes individuals according to their mental well-being. Instead, they suggest that particular behavioral configurations may be associated with a specific functional dimension of well-being.

Several explanations for the predominance of null findings are possible. First, behavioral patterns captured through smartphones may show only modest and inconsistent associations with broad self-reported well-being, particularly in generally healthy or population-based samples [96]. Second, the same observable behavior may have different meanings across individuals [97]. The sensing indicators captured the duration and frequency of app and screen use but not the content, purpose, reciprocity, or subjective quality of that use. Digital trace volume should therefore not be equated with actual exposure or lived engagement [98]. For example, 60 minutes recorded in a social media app could reflect scrolling, distressing content consumption, reciprocal communication, information seeking, content creation, or leaving an app open without sustained attention [99]. These forms of engagement may have different associations with well-being but cannot be distinguished using app duration, screen-on time, or session frequency alone [100]. Consequently, labels such as “high social media use” describe recorded behavioral volume rather than the psychological meaning or quality of the experience. This measurement gap may partly explain why the profiles showed limited differentiation in mental well-being. Future studies should combine sensing data with content-sensitive measures, experience sampling, or participant reports to address this limitation. Third, positive affect and relationship satisfaction may depend more strongly on the quality and subjective meaning of activities and interactions than on the behavioral quantities captured by the sensing variables [100]. Finally, the data were collected in July 2020, when pandemic-related restrictions, altered work and commuting arrangements, reduced opportunities for in-person activities, and increased reliance on digital communication may have constrained both behavioral variability and the associations between behavior and well-being [101]. The predominance of null findings may therefore partly reflect this unusual temporal context rather than associations that would be expected under postpandemic conditions. These possibilities should be examined directly in future studies rather than assumed to account for the null results.

The difference between the 2 extreme profiles may nevertheless provide a hypothesis for further investigation. Prior work has found that lifestyle was linked to cognitive performance [102], cognitive function [103], and cognitive impairment [104]. Using objectively collected smartphone data, the current study reconfirmed the relevance of lifestyle differences to personal functioning. Specifically, the way people organize their days—including how much they move, how often they check their phones, and how much time they spend on communication and social media apps—relates selectively to positive functioning. The “active and unplugged” profile (person-level profile 5) exhibited higher positive functioning than the “mobile and always-on social” profile (person-level profile 7). One possible explanation is that heavy social app use may fragment attention and deplete cognitive resources, undermining perceived competence and clarity [105,106], whereas physical activity may acutely enhance cognitive performance [107]. However, the present correlational data cannot determine which feature, if any, accounts for the observed difference. The contrast may also reflect unmeasured factors such as occupational demands, commuting, socioeconomic circumstances, physical health, or preexisting functioning. Therefore, interpretations should be regarded as possible explanations for future testing rather than mechanisms demonstrated by this study.

The physically active and digitally unplugged pattern (person-level profile 5) was dominated by days marked by walking/cycling and minimal phone usage (day-level profile 4). Prior work consistently found that reduced smartphone usage was associated with higher levels of well-being, possibly via less digital toxicity and greater engagement with real-world life [108-110]. In addition, the screen logs of people in this profile showed more frequent activity but shorter durations. This pattern indicates how often and for how long the phone was active, but it does not reveal whether these sessions involved purposeful task completion, reciprocal communication, passive browsing, or another form of engagement. Possibly, this combination could suggest purpose-driven interactions (brief checks/replies) rather than passive social media scrolling, which indicates a healthier phone usage style that has been shown to relate to better mental well-being [100]. Additionally, high probabilities of walking and cycling in this profile are strong indicators of better cognitive performance [107]. The relatively low levels of loudness and brightness indicate that the movement of persons in this profile occurs in calmer environments rather than noisy, attention-demanding spaces, an environment previously associated with better positive functioning [111]. Moreover, during the pandemic, greater physical activity and lower phone use may have reflected differential access to outdoor spaces, employment arrangements, caregiving responsibilities, or local restrictions rather than a stable post-pandemic lifestyle. Thus, the observed association with positive functioning should not be generalized beyond the study context without replication.

Person-level profile 7 was dominated by day-level profile 1, days with high social media app duration, above-average mobility, relatively loud and bright contexts, and frequent phone sessions, with a smaller share of day-level profile 3 (sedentary, social-heavy days with longer sessions, but fewer pickups). Social media duration was consistently high, while communication-app duration is moderate. On most days, screen frequency was high alongside moderate-to-high screen time. Variability arose on days from profile 3; lower frequency, but longer durations. Mobility and activity are above average on the dominant days from day-level profile 1, interleaved with lower-movement and social media–heavy sedentary days from day-level profile 3. In summary, person-level profile 7 comprised individuals with high recorded social media app duration, frequent phone sessions, moderate-to-high screen time, and above-average mobility. These measures indicate the volume and frequency of device activity, but do not establish sustained attention, exposure to particular content, passive use, or active social interaction. One plausible explanation is that these were long-distance commuting days on which people were heavily engaged with their smartphones, which can be exhausting [112]. In addition, mobility and digital engagement in July 2020 may have been shaped by pandemic-related arrangements. Profile 7 should therefore not be interpreted as a stable “always-on” lifestyle without replication using postpandemic data.

Taken together, only positive functioning reliably differed across 2 of 7 person-level profiles; positive affect and personal relationships may require longer observation windows or additional features (eg, social-interaction content/quality) to detect profile differences.

### Interactions of Lifestyle and Personality in Mental Well-Being

The examination of lifestyle profile×personality interactions provided no evidence that personality moderated the associations between behavioral profiles and mental well-being. A likely explanation is limited statistical power to detect moderation effects. Interaction effects are typically harder to detect than main effects, and statistical power is limited unless designs are optimized and samples are very large [113]. A priori power calculations using Cohen *f*^2^= .02 (small effect) indicated that approximately n=1261 participants would be required to achieve 80% power for the omnibus interaction test (computed with the *pwr* R package [114]), which exceeds the available sample size in the present study. Accordingly, the current findings should not be taken as strong evidence that moderation is absent; rather, they suggest that any profile×personality interactions—if present—are likely small and will require larger, confirmatory samples to detect reliably.

### Implications

Findings from this study have important implications. First, we advanced a digital phenotyping approach on mental well-being, showing that well-being may be reflected in specific combinations of daily behaviors. This approach underscores the value of person-centered analytic approaches for capturing heterogeneity in lifestyles.

Second, should predictive validity be established, this approach may have the potential to inform mental health monitoring by offering interpretable information about lifestyle configurations that may be higher risk. Unlike many previously proposed monitoring systems that rely on black-box machine learning models [115], a profile-based monitor would surface human-readable patterns (which daily profiles are trending and how often they recur) that both clinicians and users can understand. Such transparency can support shared decision-making [116], guide clinician-patient communications [117], and help users reflect on the routines contributing to their risk flags.

Third, with future experimental validation, the monitoring platforms mentioned above could support the development of just-in-time adaptive interventions (JITAIs) that aim to deliver tailored support when individuals appear to exhibit risk-like patterns, thereby potentially improving the timing of interventions [118,119]. Building on this foundation, JITAIs might use profile-informed triggers to flag candidate “windows of opportunity” and provide context-appropriate options [118]. However, these possibilities require prospective testing for efficacy, feasibility, and safety.

Fourth, rather than targeting a single behavior, prior work has proposed synergistic approaches of intervention (ie, Multiple Health Behavior Change) [120,121]. Our findings reinforce this perspective by showing that well-being could be reflected in configurations of co-occurring routines [122,123]. In practice, profile-guided interventions could coordinate a few low-friction actions (eg, a short movement cue paired with a soft screen-time nudge) during candidate high-risk days, with the goal of shifting the overall configuration. While causal efficacy remains to be established, our findings provide granularity—which behaviors tend to co-occur—that could inform the design of such multicomponent strategies proposed previously.

### Limitations and Future Directions

Despite these contributions, some limitations warrant consideration. First, our sample was drawn exclusively from an online panel of German participants during July 2020. The context of the COVID-19 pandemic may have influenced the behavioral patterns captured in the study. Public policies altered work and commuting arrangements, and reduced access to social activities may shape the ways in which daily activities were organized [124]. Consequently, some of the identified profiles may reflect adaptations to the pandemic context rather than stable and generalizable lifestyle phenotypes. The present findings should therefore not be assumed to represent general behavioral patterns. Given that lifestyle norms and their links to well-being may differ across historical periods and cultures [125], direct replications using post-pandemic data, diverse cultural contexts, and repeated measurement across different time periods are necessary to determine whether the profiles are reproducible and temporally stable. Second, the current study recruited only Android users, as our app operates exclusively on this system. Given documented differences between Android and iOS users in education, income, and extraversion [126], future research should consider platform-related selection effects when replicating the results. Third, for interpretability, we selected only a limited set of sensing features; digital-phenotyping research suggests that more comprehensive multimodal data (eg, physiological data) can yield richer lifestyle profiles and stronger links to mental health outcomes [14]. Finally, our study design was correlational and cannot establish causality. It remains unclear whether certain routines impact well-being, whether well-being levels lead to certain routines, or whether both are explained by third variables, such as age, which also differed across profiles.

In light of these limitations, the present study should be viewed as an initial step toward interpretable, digital trace–based lifestyle profiling. Accordingly, several avenues for future research follow. First, the present findings show that positive functioning is reflected in lifestyles inferred from digital traces in generally healthy populations. An important next step is to examine clinical samples and test whether they show similar lifestyle patterns to those in the general population and whether certain lifestyles indicate risk for particular mental disorders. This would provide a more complete picture of digital phenotyping and inform actionable intervention practices. Second, the current interpretation of the profiles is post hoc and speculative. To validate these profiles, future studies should use methods such as qualitative interviews or daily diary approaches, asking individuals belonging to a specific profile about their daily activities. Combined with asking them how they feel throughout the day, further work could examine dynamic fluctuations in their daily lifestyles and mood. This would allow modeling of dynamic, within-person relationships between daily patterns and well-being. Third, potential weekday–weekend differences may emerge in both behavior and well-being. Future studies could consider this by examining longer timeframes that include sufficient weekend data, which would yield insights into work–life balance and how people spend their workdays and free days in ways that reflect their well-being.

### Conclusions

This study demonstrates the feasibility of using multilevel LPA to identify interpretable day- and person-level configurations from mobile-sensing data. Evidence linking these profiles to mental well-being was limited; profiles did not differ in overall mental well-being, positive affect, or satisfying interpersonal relationships, and only 2 of the 7 profiles differed in positive functioning. No evidence of personality moderation was observed. These findings indicate that distinguishable behavioral profiles do not necessarily correspond to distinguishable levels of mental well-being. Future studies should replicate the positive-functioning contrast, examine within-person temporal associations, and establish predictive and incremental validity before considering profile-based monitoring or intervention applications.

## Supplementary material

10.2196/101850 Multimedia Appendix 1 Supplemental analyses, tables and figures.

## Abbreviations

AIC: Akaike information criterion
BFI-2-XS: 15-item short forms of the Big Five Inventory–2
BIC: Bayesian information criterion
DBSCAN: Density-Based Spatial Clustering of Applications with Noise
FIML: full information maximum-likelihood
IRB: Institutional Review Board
JITAI: just-in-time adaptive intervention
LMU Munich: Ludwig-Maximilians-Universität München
LPA: latent profile analysis
MAR: missing at random
ML-LPA: multilevel-latent profile analysis
MLR: robust maximum likelihood
SSPS: Smartphone Sensing Panel Study
STROBE: Strengthening the Reporting of Observational Studies in Epidemiology
WEMWBS: Warwick-Edinburgh Mental Well-Being Scale
WHO: World Health Organization
