# Supplementary material for: Digital Phenotyping of Lifestyle Profiles and Mental Well-Being in German Adults: Prospective Longitudinal Cohort Study
Source: J Med Internet Res. 2026 Sep 14;28:e101850. doi: 10.2196/101850 (PMC13574308; doi:10.2196/101850)
Supplement: Supplementary file 1 [file jmir-v28-e101850-s001.pdf]

Supplemental Materials

Text S1	Illustration of Latent Profile Analysis.
Analysis 1	Comparison of Retained and Excluded Participants
Table S1.	Comparison of Age Between Retained and Excluded Participants.
Table S2.	Comparison of other Demographic Characteristics Between Retained and Excluded Participants.
Ananlysis2	Comparison of the Current Sample Composition With the Initial Sampling Quotas
Table S3.	Observed and Expected Demographic Composition of the Final Analytical Sample Based on the Initial Sampling Quotas.
Table S4.	Chi-Square Goodness-of-Fit Tests Comparing the Final Analytical Sample With the Initial Sampling Quotas.
Analysis 3.	Confirmatory Factor Analysis of the WEMWBS
Table S5.	Fit indices for the one-factor and correlated three-factor models of the WEMWBS (N = 553).
Analysis 4.	Missingness test.
Table S6.	Results of Missing Completely At Random Test.
Analysis 5.	One-Level Latent Profile Analysis Using Two-Week Aggregated Values
Table S7.	Model Fit Statistics for one-level LPA Model.
Table S8.	Single-level Latent profiles of sensing variables aggregated by participants.
Table S9.	Pairwise comparisons of Positive Functioning scores across 7 personal profiles.
Analysis 6.	Well-Being Differences After Omitting the Smallest Profile.
Table S10.	Adjusted means of well-being outcomes across person-level profiles after omitting profile 2.
Analysis 7.	Demographic Differences Across Person-Level Profiles
Table S11.	Adjusted means of demographic statistics across person-level profiles.
Table S12.	Adjusted distribution of income levels across person-level profiles.
Table S13.	Adjusted distribution of educational levels across person-level profiles.
Table S14.	Pairwise comparisons of Age scores across 7 personal profiles.
Table S15.	Pairwise comparisons of distributions of education levels across 7 personal profiles.
Fig. S1	Distributions of sensing features.
Table S16.	Descriptive Statistics and Measurement Scales of the Raw Daily Sensing Features Before Transformation and Standardization.

Text S1. Illustration of Latent Profile Analysis

For person $i = 1, \dots, N$, and indicators $k = 1, \dots, K$, a latent profile $Z_i \in \{1, \dots, J\}$, it is assumed:

$$Z_i \sim Categorical(\pi_1, \dots, \pi_J), \sum_{j=1}^{J} \pi_j = 1 \tag{1}$$

$$y_{ik} \mid Z_i = j \sim N(\mu_{jk}, \sigma_{jk}^2). \tag{2}$$

The marginal density of $y_i = (y_{i1}, \dots, y_{iK})$ is

$$p(y_i) = \sum_{j=1}^{J} \pi_j \prod_{k=1}^{K} N(y_{ik} \mid \mu_{jk}, \sigma_{jk}^2), \tag{3}$$

which means how plausible y_i would be if person i were in profile j. Parameters $(\pi_j, \mu_{jk}, \sigma_{jk}^2)$ are estimated by maximum likelihood with multiple random starts to mitigate local optima. Individuals are characterized by posterior class probabilities

$$\tau_{ij} = P(Z_i = j \mid y_i) = \frac{\pi_j \prod_{k=1}^{K} \mathcal{N}(y_{ik} | \mu_{jk}, \sigma_{jk}^2)}{\sum_{\ell=1}^{J} \pi_\ell \prod_{k=1}^{K} \mathcal{N}(y_{ik} | \mu_{\ell k}, \sigma_{\ell k}^2)}. \tag{4}$$

In the two-level setting, individuals $i = 1, \dots, N$ are nested within clusters $u = 1, \dots, U$, Level-1 latent profiles $Z_{iu} \in \{1, \dots, J\}$ capture within-cluster heterogeneity in the indicators y_{iku} , while Level-2 latent profiles $B_u \in \{1, \dots, B\}$ capture between-cluster heterogeneity in the relative frequencies of the Level-1 profiles. As fomular (2), it is assumed that:

$$y_{iku} \mid Z_{iu} = j \quad \sim N(\mu_{jk}, \sigma_{jk}^2), k = 1, \dots, K, \; j = 1, \dots, J, \tag{5}$$

The Level-2 classes have mixing proportions $B_u \sim Categorical(\rho_1, \dots, \rho_B), \sum_{b=1}^{B} \rho_b = 1$, Conditional on $B_u = b$, the Level-1 class of person i in cluster u follows $Z_{iu} \mid B_u = b \sim Categorical(\pi_{1|b}, \dots, \pi_{J|b})$, parameterized by multinomial logits:

$$lo\,g \frac{\pi_{j|b}}{\pi_{J|b}} = \alpha_j + \beta_{jb}, j = 1, \dots, J-1, \;\; b = 1, \dots, B-1, \tag{6}$$

$$\pi_{j|b} = \frac{exp(\alpha_j + \beta_{jb})}{\sum_{\ell=1}^{J} exp(\alpha_\ell + \beta_{\ell b})} \tag{7}$$

Level-1 profile proportions are mixtures over Level-2 profiles: $\bar{\pi}_j = \sum_{b=1}^{B} \rho_b \, \pi_{j|b}, j = 1, \dots, J.$ Let $y_u = \{y_{iku}: i \in u,\ k = 1, \dots, K\}$, The marginal likelihood for class u is

$$p(y_u) = \sum_{b=1}^{B} \rho_b \prod_{i \in u} \{\sum_{j=1}^{J} \pi_{j|b} \prod_{k=1}^{K} N\,(y_{iku} \mid \mu_{jk}, \sigma_{jk}^2)\}, \tag{7}$$

Parameters $\left(\rho_b, \pi_{j|b}, \mu_{jk}, \sigma_{jk}^2\right)$ are estimated by maximum likelihood. Posterior probabilities at both levels follow by Bayes' rules,

$$P(\,B_u = b \mid y_u\,) \propto \rho_b \prod_{i \in u} \{\sum_{j=1}^{J} \pi_{j|b} \prod_{k=1}^{K} N\,(y_{iku} \mid \mu_{jk}, \sigma_{jk}^2)\} \tag{8}$$

$$P(Z_{iu} = j \mid B_u = b, y_u) \propto \pi_{j|b} \prod_{k=1}^{K} N\,(y_{iku} \mid \mu_{jk}, \sigma_{jk}^2). \tag{9}$$

Supplemental Analysis 1. Comparison of Retained and Excluded Participants

We conducted an attrition analysis to examine whether participants who were retained in the analytic sample differed from those who were excluded after applying the requirement of at least 8 valid days of data across the 14-day assessment period. The retained group consisted of 553 participants, whereas the excluded group consisted of 59 participants.

The groups were compared with respect to age and selected demographic characteristics. Age was compared using an independent-samples Welch's t test. Gender, education, income, religious affiliation, and relationship status were compared using Pearson's chi-square tests. To account for multiple comparisons across the five categorical variables, p values were adjusted using the Holm correction.

**Variable**	**Retained, M (SD)**	**Excluded, M (SD)**	**t**	**p**
Age	42.12 (12.89)	39.96 (13.28)	0.684	0.496
Well-Being	3.5 (0.67)	3.45 (0.79)	0.597	0.552
Positive Affect	3.31 (0.77)	3.23 (0.86)	0.765	0.446
Positive Functionality	3.76 (0.71)	3.77 (0.79)	-0.059	0.953
Relation Satisfaction	3.53 (0.76)	3.45 (0.91)	0.771	0.442

Table S1. Comparison of Age and Well-Being Between Retained (n = 553) and Excluded Participants (n = 83). Group differences were examined using an independent-samples t test, which does not assume equal variances. M = mean; SD = standard deviation.

As shown in Table S1, retained participants did not differ significantly in age and the well-being outcomes from excluded participants. Similarly, the chi-square tests presented in Table S2 indicated no significant group differences in gender, education, income, religious affiliation, or relationship status. Overall, the results provided no evidence of systematic demographic differences between participants who were retained and those who were excluded based on the valid-day criterion.

**Variable**	χ^2	**df**	**p**
Gender	0	1	0.984
Education	2.37	5	0.795
Income	13.4	11	0.267
Relationship status	0.716	1	0.397

Religion	4.12	5	0.532

Table S2. Comparison of other Demographic Characteristics Between Retained (n = 553) and Excluded Participants (n = 83). Group differences were examined using Pearson's chi-square tests of independence. χ^2 = Pearson's chi-square statistic; df = degrees of freedom.

Supplemental Ananlysis2. Comparison of the Current Sample Composition With the Initial Sampling Quotas

We compared the demographic composition specified in the initial sampling design—that is, the quota composition of participants selected to receive the questionnaires—with the composition of the final analytical sample using chi-square goodness-of-fit tests.

**Variable**	**Category**	**Observed n**	**Expected n**	**Observed percent**	**Quota percent**
Gender	Male	274	248	55.4	50
Gender	Female	221	248	44.6	50
Age	18–29	98	111	19.8	22.5
Age	30–39	127	99	25.7	20
Age	40–49	94	99	19	20
Age	50–65	176	186	35.6	37.5
Education	No school leaving certificate / primary school equivalent	72	137	18.4	35
Education	Secondary school / equivalent	172	122	43.9	31.2
Education	High school / equivalent	148	132	37.8	33.8
Income	Less than €1,500	214	161	43.2	32.5
Income	€1,500–€2,500	189	198	38.2	40
Income	More than €2,500	92	136	18.6	27.5
Religion	No religion	241	185	48.8	37.5
Religion	Christianity	245	266	49.6	53.8
Religion	Others	8	43.2	1.62	8.75
In a relationship	Yes	329	328	66.5	66.2
Note in a relationship	No	166	167	33.5	33.8

Table S3. Observed and Expected Demographic Composition of the Final Analytical Sample Based on the Initial Sampling Quotas. Observed values refer to the demographic composition of the final analytical sample. Expected values were calculated from the demographic quota specified in the initial sampling design for participants selected to receive the questionnaires and were rescaled to the valid sample size for each variable. Percentages are based on nonmissing responses. For education, respondents reporting a university degree or doctorate were excluded because these categories were not directly represented in the original quota classification. For religion,

respondents who selected “prefer not to answer” were excluded. The original relationship-status quota categories of married/registered partnership and in a relationship were combined into a single “Yes” category to correspond with the binary measure available in the final sample. Expected counts may not sum exactly to the valid sample size because of rounding.

Variable	χ^2	df	p	p_adjusted	Cohen’s w
Gender	5.67	1	0.017	0.103	0.107
Age	10.3	3	0.016	0.098	0.144
Education	52.8	2	< .001	< .001	0.367
Income	32.3	2	< .001	< .001	0.255
Confession	47.1	2	< .001	< .001	0.309
Relationship status	0.0102	1	0.92	1.00	0.005

Table S4. Chi-Square Goodness-of-Fit Tests Comparing the Final Analytical Sample With the Initial Sampling Quotas. Chi-square goodness-of-fit tests compared the demographic composition of the final analytical sample with the quota composition specified in the initial sampling design for participants selected to receive the questionnaires. The reported adjusted p values were obtained using the Bonferroni correction across the six demographic comparisons. Cohen’s w is reported as the effect-size measure, with values of approximately .10, .30, and .50 conventionally indicating small, medium, and large effects, respectively. After correction, gender, age, and relationship status did not differ significantly from the designed quota, whereas education, income, and religion did.

After adjustment, the final sample did not differ significantly from the designed quota in terms of gender, age, or relationship status. Significant differences were observed for education, income, and religious affiliation. However, although these differences were statistically significant, the effect sizes were small to moderate (w = .255–.367), indicating that the deviations were meaningful but not large.

Supplemental Analysis 3. Confirmatory Factor Analysis of the WEMWBS

To examine the factorial validity of the Warwick–Edinburgh Mental Well-being Scale (WEMWBS) in the present sample, we conducted confirmatory factor analyses using the 14 ordinal items. Models were estimated using the robust weighted least squares estimator with mean- and variance-adjusted test statistics (WLSMV). We compared a one-factor model, in which all items loaded on a general mental-well-being factor, with a correlated three-factor model comprising positive affect, positive functioning, and satisfying interpersonal relationships.

Model	Chi_square	df	p	CFI	TLI	RMSEA	RMSEA_lower	RMSEA_upper	SRMR
One-factor	913	77	0	0.944	0.933	0.14	0.132	0.148	0.068
Correlated three-factor	552	74	0	0.968	0.96	0.108	0.100	0.117	0.051

Table S5. Fit indices for the one-factor and correlated three-factor models of the WEMWBS (N = 553). WEMWBS = Warwick–Edinburgh Mental Well-being Scale; CFI = comparative fit index; TLI = Tucker–Lewis index; RMSEA = root mean square error of approximation; SRMR = standardized root mean square residual. Models were estimated using WLSMV for ordinal indicators.

As shown in Table S5, the correlated three-factor model demonstrated better fit than the one-factor model. The three-factor model had higher CFI and TLI values and lower RMSEA and SRMR values. Specifically, the correlated three-factor model showed good fit according to the CFI, TLI, and SRMR, although its RMSEA remained above conventional thresholds. Thus, the findings provide relative support for distinguishing the three domains, while also indicating that the factor structure was not uniformly well fitting across all fit indices. Accordingly, the overall WEMWBS score was retained as the primary measure of mental well-being, and the three domain scores were treated as secondary outcomes.

Supplemental Analysis 4. Missingness test.

Missingness was examined using logistic missingness-indicator models. For each incomplete variable, a binary indicator was created, coded 1 when the observation was missing and 0 when it was observed. The missingness indicator was regressed on six fully observed day-level variables. These predictors were centered around each participant's mean to isolate within-person variation [1]. Because repeated observations were nested within participants, statistical inference was based on participant-clustered robust covariance estimates [2]. A joint Wald test evaluated whether the within-person predictors collectively predicted missingness. A statistically significant test was interpreted as evidence inconsistent with a within-person missing-completely-at-random mechanism. Nonsignificant tests were interpreted as an absence of detected evidence against MCAR, rather than proof that the variable was MCAR. Missing-data mechanisms were conceptualized according to Rubin's (1976) framework [3].

**Variable**	**Missing, n (%)**	**Wald χ^2**	**df**	**p**	**Interpretation**
Distance	1,329 (17.4%)	40.10	6	< .001	Evidence against within-person MCAR
Gyration ratio	1,514 (19.8%)	38.80	6	< .001	Evidence against within-person MCAR
Brightness	436 (5.7%)	1.93	6	.926	No evidence against within-person MCAR
Loudness	106 (1.4%)	7.14	6	.309	No evidence against within-person MCAR

Table S6. Results of Missing Completely At Random Test.

Distance was missing for 1,329 of 7,635 measurement occasions (17.4%). Its missingness was jointly associated with the six within-person predictors, Wald χ^2 (6) = 40.10, $p<.001$. Gyration ratio was missing for 1,514 occasions (19.8%), and its missingness was also associated with the within-person predictors, Wald χ^2 (6) = 38.80, $p<.001$. These findings provided evidence inconsistent with within-person MCAR for distance and gyration ratio. Brightness was missing for 436 occasions (5.7%), but the joint test was nonsignificant, Wald χ^2 (6) = 1.93, $p=.926$. Loudness was missing for 106 occasions (1.4%), and its joint test was also nonsignificant, Wald χ^2 (6) = 7.14, $p=.309$. Thus, no evidence against within-person MCAR was detected for brightness or loudness

using the included predictors. These nonsignificant findings do not establish that the variables were MCAR.

Supplemental Analysis 5. One-Level Latent Profile Analysis Using Two-Week Aggregated Values

We calculated each participant's average value for each sensing variable across the two-week observation period and then conducted a one-level latent profile analysis. The results are presented below.

Class (L1)	BIC	Sample-size-adjusted BIC	∆BIC	LL	AIC	∆AIC	Entropy	Size of smallest P	BLRT_p
1	15452.1851600304	15388.696285668		-7662.939	15365.878		1	1	
2	14605.3820980472	14506.9743427854	846.803062	-7204.803	14471.606	894.272	0.881	0.30018	0
3	14265.0550360639	14131.7283999027	340.327062	-6999.905	14083.81	387.796	0.92	0.03797	0
4	14115.8799740807	13947.6344570201	149.175062	-6890.583	13887.166	196.644	0.859	0.03797	0
5	13971.3829120974	13768.2185141375	144.497062	-6783.6	13695.2	191.966	0.821	0.03797	0
6	13892.6078501142	13654.5245712549	78.77506198	-6709.478	13568.956	126.244	0.857	0.03617	0
7	13882.4927881309	13609.4906283723	10.11506198	-6669.686	13511.372	57.584	0.835	0.03797	0
**8**	**13802.2737261477**	**13494.3526854897**	**80.21906198**	**-6594.842**	**13383.684**	**127.688**	**0.876**	**0.03617**	**0**
9	13830.0706641644	13487.230742607	-27.79693802	-6574.006	13364.012	19.672	0.863	0.02532	0.1304
10	13768.9136021812	13391.1547997244	61.15706198	-6508.693	13255.386	108.626	0.884	0.00542	0

Table S7. Model Fit Statistics for one-level LPA Model. BIC = Bayesian Information Criterion; AIC = Akaike Information Criterion; LL = log-likelihood. P = Profile(s); Bold numbers show best profile solution. Lower BIC and AIC values indicate better model fit. However, these indices may continue to decrease as additional latent classes are extracted. In such cases, the optimal solution is typically identified at the point where further decreases begin to level off.

Profile	Communication	Social Media	Distance	Gyratio	Walking	On-Bicycle	Loudness	Brightness	Screen Count	Screen Time	n
1	-0.352	-0.458	0.502	0.517	0.375	0.389	0.131	0.243	-0.102	-0.174	168
2	0.643	0.726	0.445	0.427	0.351	0.415	0.149	0.257	0.723	0.494	161
3	0.103	-0.058	-1.957	-1.981	0.014	-0.214	0.147	0.245	0.17	-0.085	89
4	-0.091	0.085	0.538	0.568	0.623	-0.387	-7.075	-0.187	0.482	-0.237	10
5	0.049	-0.049	-0.238	-0.178	-0.159	-0.165	0.134	-4.097	-0.319	-0.032	30
6	-1.314	-0.882	0.48	0.488	-0.236	0.052	0.115	0.222	-1.253	-1.643	23
7	-0.802	-0.383	0.474	0.47	-1.636	-1.461	0.126	0.23	-0.879	-0.511	55
8	1.359	0.366	-1.005	-0.962	-1.63	-1.629	-0.113	0.181	-1.736	2.05	17

Table S8. Single-level Latent profiles of sensing variables aggregated by participants. Communication = Communication Apps Usage Duration; Social Media = Social Media Apps Usage Duration. n = number of participants.

Our empirical comparison supports the necessity of the two-level LPA because the single-level model and the multilevel model answer substantively different questions.

The single-level LPA classifies people using their average sensing values across two weeks. Consequently, it mainly identifies people with generally high or low average activity. For example, one-level Profile 2 is broadly high in communication, social media, mobility, screen count, and screen time, whereas Profile 7 is low in walking, cycling, communication, and screen activity. Several small profiles are also largely defined by extreme person-level averages, such as very low loudness in Profile 4, very low brightness in Profile 5, and unusually high screen time combined with low mobility in Profile 8. The two-level model retains the organization of behavior within individual days before summarizing people. This reveals several distinctions that are obscured by person-level averages:

First, the same overall behavior occurs in qualitatively different daily contexts. High social-media use appears in both day-level Profile 1 and day-level Profile 3. However, Profile 1 combines high social-media use with above-average walking, cycling, mobility, screen checking, and communication, whereas Profile 3 combines high social-media use with very low mobility, walking, and cycling. A person-level mean of social-media use cannot distinguish these two behavioral contexts.

Second, the two-level analysis differentiates several forms of active days. Day-level Profile 1 reflects active and digitally engaged days; Profile 4 reflects active but digitally disengaged days; Profile 5 reflects active days with low social-media use but moderate screen checking. In the single-level model, these configurations are compressed into average levels and may be interpreted merely as more or less active lifestyles.

Third, the multilevel model distinguishes stable lifestyles from heterogeneous lifestyles. Person-level Profile 6 consists almost entirely of day-level Profile 5 days, with 92.3% of its observations assigned to that pattern. Person-level Profile 7 similarly consists predominantly of day-level Profile 1 days, at 83.7%. In contrast, person-level Profile 2 is composed mainly of two uncommon day types, day-level

Profiles 6 and 8, accounting for 31.0% and 37.9% of its days. Thus, the two-level model captures whether a person repeatedly follows one routine or alternates among several distinct routines. This information is necessarily lost when all days are averaged before profile estimation.

Fourth, the multilevel approach handles rare behavioral states more meaningfully. In the single-level solution, very small person-level profiles are formed around extreme averaged values, including profiles with n=10, n=17, and n=23. In the two-level model, unusual patterns such as very low loudness and brightness are represented as rare day-level profiles, comprising 1.3% and 1.8% of all days, and are then incorporated into broader person-level lifestyle compositions. This avoids forcing every person who experiences some unusual days into a small and potentially unstable person-level class.

In conclusion, the two-level model does not merely reproduce the single-level profiles with more complexity. It changes the unit and meaning of the analysis.

	Estimate	SE	*Z*	*p*	p-adjusted
P1-P2	-0.06739	0.19142	-0.3521	0.7247856	1
P1-P3	-0.01776	0.13963	-0.1272	0.8988133	1
P1-P4	-0.09824	0.16579	-0.5926	0.5534606	1
P1-P5	-0.19437	0.1397	-1.3913	0.1641268	1
P1-P6	-0.04545	0.13559	-0.3352	0.7374755	1
P1-P7	0.13574	0.13594	0.9986	0.3179899	1
P2-P3	0.04964	0.16553	0.2999	0.7642845	1
P2-P4	-0.03085	0.18725	-0.1648	0.8691345	1
P2-P5	-0.12698	0.16302	-0.7789	0.4360134	1
P2-P6	0.02194	0.16186	0.1356	0.8921569	1
P2-P7	0.20314	0.16244	1.2506	0.2110937	1
P3-P4	-0.08049	0.13478	-0.5972	0.5503957	1
P3-P5	-0.17662	0.09739	-1.8135	0.0697577	1
P3-P6	-0.02769	0.09585	-0.2889	0.7726552	1
P3-P7	0.1535	0.09751	1.5742	0.1154401	1
P4-P5	-0.09613	0.13079	-0.735	0.4623384	1
P4-P6	0.0528	0.12933	0.4082	0.6831125	1
P4-P7	0.23399	0.13209	1.7714	0.0764927	1
P5-P6	0.14893	0.09193	1.6199	0.1052495	1
P5-P7	0.33012	0.09181	3.5955	0.0003238	0.006799
P6-P7	0.18119	0.08973	2.0192	0.0434659	0.869318

Table S9. Pairwise comparisons of Positive Functioning scores across 7 personal profiles. P = Personal Profile. Multiple comparisons were controlled by adjusting *p* values with the Holm (sequential Bonferroni) procedure to control the family-wise error rate.

Supplemental Analysis 6. Well-Being Differences After Omitting the Smallest Profile.

Person-Level Profile	Mental Well-being	Positive Affect	Positive Functioning	Personal Relationship
1 (indoor, low engagement)	3.41 (0.11)	3.16 (0.12)	3.74 (0.12)	3.46 (0.12)
3 (mobile, mixed-level social)	3.54 (0.07)	3.37 (0.08)	3.76 (0.07)	3.57 (0.08)
4 (social-heavy, sedentary-lean)	3.47 (0.11)	3.22 (0.12)	3.84 (0.11)	3.47 (0.12)
5 (physically active & unplugged)	3.54 (0.07)	3.33 (0.08)	3.93 (0.07)	3.48 (0.08)
6 (commute-style, bursty use)	3.55 (0.06)	3.38 (0.07)	3.79 (0.06)	3.56 (0.08)
7 (mobile & always-on social)	3.45 (0.06)	3.28 (0.07)	3.60 (0.06)	3.55 (0.06)
χ^2 ($df = 5$)	2.75	3.94	13.35*	1.56

Table S10. Adjusted means of well-being outcomes across person-level profiles after omitting profile 2. Adjusted mean refers to the mean value accounting for classification error in profile assignment. Values are means with standard errors in parentheses. χ^2 tests ($df = 6$) compare profiles; *$P < .05$.

After omitting the smallest profile (Profile 2), the pattern of results remained consistent with that of the original analysis. Specifically, a significant overall difference across profiles was observed only for positive functioning, whereas the differences in mental well-being, positive affect, and personal relationships remained nonsignificant. These findings indicate that the original results were robust to the exclusion of the smallest profile.

Supplemental Analysis 7. Demographic Differences Across Person-Level Profiles

We examined whether the seven person-level profiles differed in age, gender, relationship status, educational level, and income. All demographic estimates were adjusted for classification uncertainty in profile membership. For continuous and binary demographic variables, adjusted means or proportions were compared across profiles using omnibus Wald tests. For categorical variables, the complete distributions of educational attainment and income were compared across profiles. Pairwise comparisons were adjusted using the Holm sequential Bonferroni procedure to control the family-wise error rate.

Person-Level Profile	Age	Gender (Female %)	Relationship (In-a-relationship %)
1 (indoor, low engagement)	48.05 (1.69)	0.50 (0.07)	0.64 (0.07)
2 (indoor, deep use)	45.71 (3.02)	0.54 (0.10)	0.58 (0.10)
3 (mobile, mixed-level social)	41.01 (1.40)	0.46 (0.06)	0.66 (0.05)
4 (social-heavy, sedentary-lean)	46.25 (2.11)	0.50 (0.08)	0.61 (0.08)
5 (physically active & unplugged)	47.75 (1.36)	0.31 (0.05)	0.64 (0.05)
6 (commute-style, bursty use)	40.99 (1.26)	0.39 (0.05)	0.67 (0.05)
7 (mobile & always-on social)	35.38 (1.05)	0.51 (0.05)	0.72 (0.04)
χ^2 (*df* = 6)	78.62***	11.99	3.19

Table S11. Adjusted means of demographic statistics across person-level profiles.

Age differed significantly across the seven profiles, Wald $\chi^2(6) = 78.62$, $p < .001$. Profile 7, characterized by mobile and always-on social behavior, was the youngest on average (M = 35.38, SE = 1.05). Profiles 3 and 6 also had relatively low mean ages, at 41.01 and 40.99 years, respectively. In contrast, Profiles 1, 4, and 5 had higher mean ages, ranging from 46.25 to 48.05 years.

The proportion of female participants did not differ significantly across profiles, Wald $\chi^2(6) = 11.99$, $p > .05$. Female representation ranged from 31% in Profile 5 to 54% in Profile 2. Relationship status also did not differ significantly across profiles, Wald $\chi^2(6) = 3.19$, $p > .05$. The estimated proportion of participants in a relationship ranged from 58% in Profile 2 to 72% in Profile 7.

Person-Level Profile	Lower secondary or less	Upper secondary	Technical college entrance	University degree or higher
1	0.1696	0.3855	0.3205	0.12442
2	0.125	0.5	0.3333	0.04163
3	0.1246	0.4149	0.2094	0.25113
4	0.2332	0.244	0.3432	0.17965
5	0.1736	0.2432	0.3348	0.2484
6	0.1226	0.3959	0.2572	0.22427
7	0.1199	0.3307	0.3317	0.21778
χ^2 (*df* = 18)			33.22*	

Table S12. Adjusted distribution of income levels across person-level profiles.

Person-Level Profile	Below 1000	1000 - 1999	2000 - 2999	3000 or more
1	0.4265	0.2392	0.2034	0.13099
2	0.2499	0.4168	0.2083	0.125
3	0.2485	0.4014	0.2504	0.09976
4	0.4151	0.3159	0.1299	0.13907
5	0.2455	0.3209	0.2851	0.14857
6	0.201	0.3832	0.2696	0.14628
7	0.3142	0.3548	0.2511	0.07993
χ^2 (*df* = 18)			20.44	

Table S13. Adjusted distribution of educational levels across person-level profiles.

Income distributions did not differ significantly across profiles, Wald $\chi^2(18) = 20.44$, $p > .05$. Although some descriptive variation was evident—for example, Profiles 1 and 4 had relatively high probabilities of reporting income below 1,000, whereas Profiles 5 and 6 had relatively higher probabilities of reporting income of 2,000 or above—these differences did not provide evidence of an overall association between income and profile membership.

	Estimate	SE	*Z*	*p*	p-adjusted
P1-P2	2.34269	3.455	0.6781	4.98E-01	1
P1-P3	7.03783	2.191	3.212	1.32E-03	0.0171
P1-P4	1.80432	2.718	0.6639	5.07E-01	1
P1-P5	0.29947	2.224	0.1347	8.93E-01	1
P1-P6	7.06264	2.112	3.3442	8.25E-04	0.0124
P1-P7	12.67117	1.984	6.3871	1.69E-10	0.000
P2-P3	4.69514	3.324	1.4125	1.58E-01	1
P2-P4	-0.53837	3.679	-0.1463	8.84E-01	1
P2-P5	-2.04322	3.307	-0.6179	5.37E-01	1
P2-P6	4.71995	3.267	1.4447	1.49E-01	1
P2-P7	10.32848	3.191	3.2365	1.21E-03	0.0169
P3-P4	-5.2335	2.55	-2.0526	4.01E-02	0.401
P3-P5	-6.73836	1.955	-3.4467	5.68E-04	0.00965
P3-P6	0.02481	1.895	0.0131	9.90E-01	1.00
P3-P7	5.63335	1.768	3.1859	1.44E-03	0.0173
P4-P5	-1.50485	2.509	-0.5998	5.49E-01	1.00
P4-P6	5.25832	2.456	2.1413	3.23E-02	0.355
P4-P7	10.86685	2.389	4.5488	5.40E-06	0.000103
P5-P6	6.76317	1.878	3.6015	3.16E-04	0.00569
P5-P7	12.3717	1.715	7.2157	5.37E-13	0.000
P6-P7	5.60853	1.637	3.4271	6.10E-04	0.00976

Table S14. Pairwise comparisons of Age scores across 7 personal profiles. P = Personal Profile. Multiple comparisons were controlled by adjusting *p* values with the Holm (sequential Bonferroni) procedure to control the family-wise error rate.

Holm-adjusted pairwise comparisons indicated that Profile 7 was significantly younger than Profiles 1, 2, 3, 4, 5, and 6. Profile 3 was significantly younger than Profiles 1 and 5, whereas Profile 6 was significantly younger than Profiles 1 and 5. Profiles 3 and 6 did not differ significantly from one another. The remaining pairwise age differences were not significant after adjustment for multiple comparisons.

Educational level differed significantly across the profiles, Wald $\chi^2(18) = 33.22$, $p = .016$. Descriptively, Profile 2 had the highest probability of upper-secondary education (.500) and the lowest probability of having a university degree or higher (.042). Profiles 3, 5, 6, and 7 showed comparatively higher probabilities of having a university degree or higher. Follow-up pairwise Wald tests compared the complete distribution of educational attainment between each pair of

profiles rather than comparing category-specific odds ratios. The largest difference was observed between Profiles 2 and 5, $\chi^2(3) = 13.21$, unadjusted $p = .004$. However, this comparison did not remain significant after Holm adjustment, adjusted $p = .088$. No pairwise comparison of educational distributions was significant after correction for multiple testing.

	Estimate	df	*p*	p-adjusted
P1-P2	2.2477	3	0.52262	1
P1-P3	4.431	3	0.218525	1
P1-P4	2.2576	3	0.52069	1
P1-P5	4.2095	3	0.23971	1
P1-P6	2.6636	3	0.446453	1
P1-P7	2.7009	3	0.440078	1
P2-P3	10.9402	3	0.012054	0.24108
P2-P4	7.1861	3	0.066196	1
P2-P5	13.2081	3	0.004208	0.08836
P2-P6	9.07	3	0.028375	0.51074
P2-P7	10.6687	3	0.013659	0.25952
P3-P4	6.4721	3	0.090767	1
P3-P5	6.42	3	0.092872	1
P3-P6	0.5419	3	0.909585	1
P3-P7	3.7309	3	0.292031	1
P4-P5	1.0585	3	0.787097	1
P4-P6	4.8478	3	0.183289	1
P4-P7	2.907	3	0.406193	1
P5-P6	4.583	3	0.205003	1
P5-P7	2.3767	3	0.497984	1
P6-P7	1.5495	3	0.670887	1

Table S15. Pairwise comparisons of distributions of education levels across 7 personal profiles. P = Personal Profile. Multiple comparisons were controlled by adjusting *p* values with the Holm (sequential Bonferroni) procedure to control the family-wise error rate.

Overall, the profiles were differentiated most clearly by age. Educational level also showed a significant overall association with profile membership, although no individual pairwise profile comparison remained significant after multiplicity correction. Gender composition, relationship status, and income distribution did not differ significantly across the seven profiles.

Fig. S1. Distributions of sensing features

A. Distributions before Box-Cox transformation

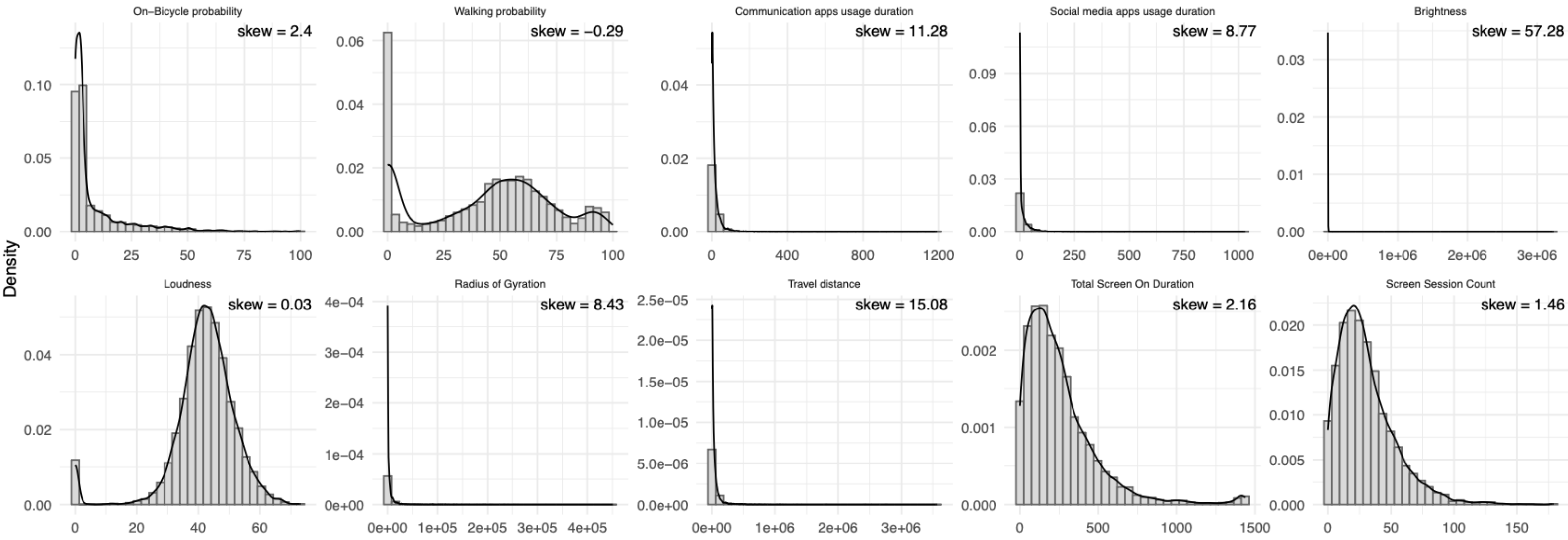

B. Distributions after Box-Cox transformation

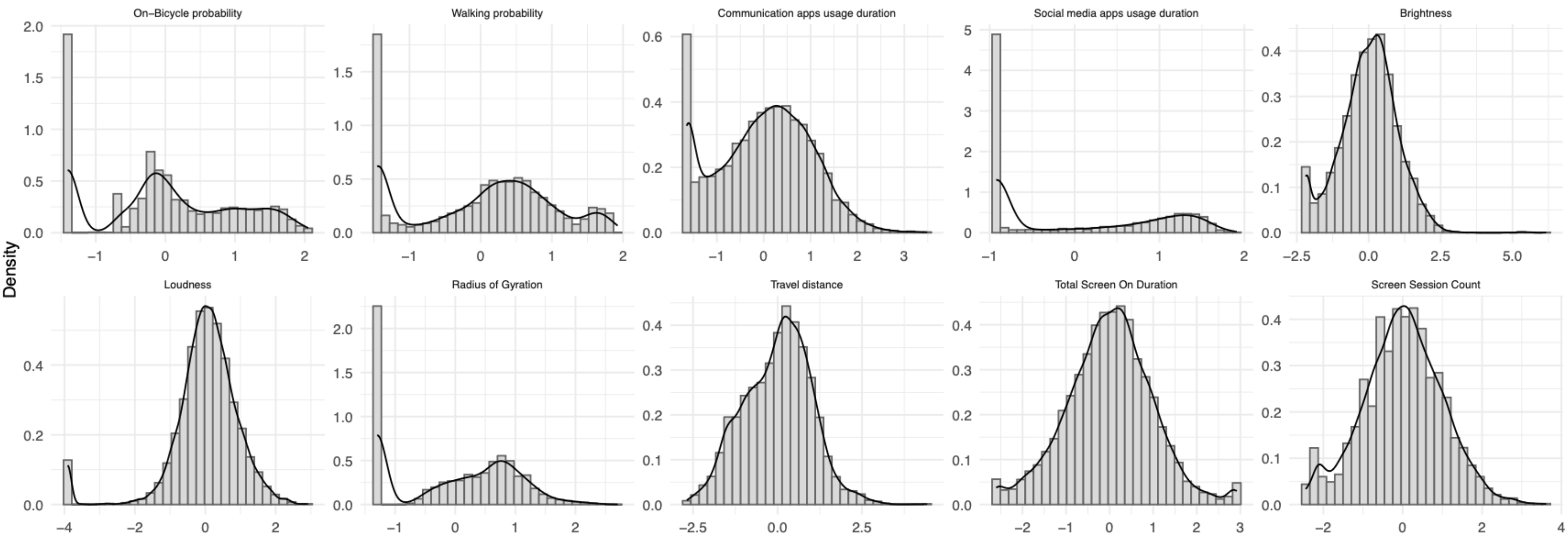

Feature	Scale	Mean	SD	Minimum	Q1	Median	Q3	Maximum	Skewness
On-bicycle probability	Percentage, 0-100	9.05	15.26	0	1	2.62	9.79	100	2.75
Walking probability	Percentage, 0-100	43.02	29.6	0	10.5	48.71	63.79	100	-0.17
Communication app usage duration	Minutes per day	19.3	46.29	0	2.12	7.98	20.89	1191.68	11.64
Social media app usage duration	Minutes per day	14.41	41.29	0	0	0.29	14.6	1030.6	10.92
Brightness	Lux	1251.14	44676.38	0	12.25	39.47	109.78	3234173.07	58.29
Loudness	Decibels (dB)	41.84	10.71	0	37.65	42.61	47.78	73.11	-1.59
Radius of gyration	Meters per day	4920.79	17555.59	0	0	490.1	3452.4	451014.87	9.98
Travel distance	Meters per day	42337.96	115910.29	0	2718.45	14946.64	43553.23	3568290.41	15.08
Screen-on duration	Minutes per day	258.45	234.53	0	100.25	201.54	341.99	1437.74	2.16
Screen session count	Number of sessions per day	30.29	22.51	0	14	26	41	179	1.45

Table S16. Descriptive Statistics and Measurement Scales of the Raw Daily Sensing Features Before Transformation and Standardization. Duration variables are reported in minutes per participant-day. Travel distance and radius of gyration are reported in meters. Walking and on-bicycle indicators range from 0 to 100 and represent the estimated percentage probability of each activity. Screen session count represents the number of screen sessions per day. The multilevel latent profile analysis was conducted using Box–Cox-transformed and z-standardized indicators.

References:

1. Enders CK, Tofighi D. Centering predictor variables in cross-sectional multilevel models: A new look at an old issue. Psychol Methods US: American Psychological Association; 2007;12(2):121–138. doi: 10.1037/1082-989X.12.2.121

2. Zeileis A, Köll S, Graham N. Various Versatile Variances: An Object-Oriented Implementation of Clustered Covariances in *R*. J Stat Softw 2020;95(1). doi: 10.18637/jss.v095.i01

3. Rubin DB. Inference and missing data. Biometrika 1976;63(3):581–592. doi: 10.1093/biomet/63.3.581
